# Integrated transcriptomic and functional analysis reveals selective cytotoxicity and HPV oncogene suppression induced by cinnamon essential oil in cervical cancer cells

**DOI:** 10.1186/s12906-026-05471-7

**Published:** 2026-07-28

**Authors:** Ruben Piña-Cruz, Andrea Molina-Pineda, Sayma Vizcarra-Ramos, Jesús García-Gómez, Mariel García-Chagollán, Rodolfo Hernández-Gutiérrez, Luis Felipe Jave-Suarez, Adriana Aguilar-Lemarroy

**Affiliations:** 1 Programa de Doctorado en Ciencias en Biología Molecular en Medicina, Centro Universitario de Ciencias de la Salud (CUCS), Universidad de Guadalajara, Guadalajara, Jalisco 44340 Mexico; 2 División de Inmunología, Centro de Investigación Biomédica de Occidente (CIBO), Instituto Mexicano del Seguro Social (IMSS), Guadalajara, Jalisco 44340 Mexico; 3 Centro de Investigación y Asistencia en Tecnología y Diseño del Estado de Jalisco A.C. (CIATEJ), Guadalajara, 44270 Mexico; 4 Programa de Doctorado en Ciencias Biomédicas, Centro Universitario de Ciencias de la Salud (CUCS), Universidad de Guadalajara, Guadalajara, Jalisco 44340 Mexico; 5 Instituto de Investigación en Ciencias Biomédicas, Centro Universitario de Ciencias de la Salud, Universidad de Guadalajara, Guadalajara, 44340 Mexico; 6 Unidad de Investigación Epidemiológica y en Servicios de Salud, Instituto Mexicano del Seguro Social (IMSS), Guadalajara, Jalisco 44340 Mexico

**Keywords:** *Cinnamon* essential oil, *Cinnamomum zeylanicum*, Cervical cancer, Apoptosis, Mitochondrial pathway, HPV E6/E7

## Abstract

**Background:**

Cervical cancer (CC) remains a major cause of cancer-related mortality among women worldwide, primarily driven by persistent infection with high-risk human papillomavirus (HPV) types. Given that natural products with selective antitumour properties offer potential for novel therapies, we evaluated the cytotoxic, antiproliferative, and molecular effects of *cinnamon* essential oil (CEO) on HPV-positive cervical cancer cell lines (HeLa and SiHa) and nontumorigenic keratinocytes (HaCaT).

**Methods:**

Cells were exposed to a range of dilutions of CEO. Cell viability was determined by WST-1 and sulforhodamine B assays. Apoptosis was assessed using Annexin V/PI staining, caspase-3/8/9 activation, and mitochondrial membrane potential (Δψm) loss by flow cytometry. Proliferation was measured using EdU incorporation, and senescence was measured by β-galactosidase activity. Genome-wide transcriptional changes and HPV E6/E7 oncogene expression were analysed by RNA-Seq and validated with qPCR.

**Results:**

CEO induced dose-dependent cytotoxicity and inhibited proliferation in cervical cancer cells, while HaCaT cells were significantly more resistant. Apoptosis was evidenced by Annexin V/PI positivity, activation of caspases -8, -9, and -3, and significant loss of mitochondrial membrane potential. No significant senescence induction was observed. Additionally, CEO also significantly downregulated HPV *E6*/*E7* oncogene expression in HeLa and SiHa cells. Transcriptomic analysis revealed broad differential gene expression, including upregulation of stress and apoptosis-related genes and downregulation of proliferation, oxidative phosphorylation, and viral-related pathways.

**Conclusion:**

CEO exerts selective cytotoxic and antiproliferative effects on cervical cancer cells by triggering mitochondria-mediated apoptosis and suppressing HPV oncogenes. These findings support CEO as a potential source of bioactive compounds for complementary strategies against HPV-related malignancies.

**Graphical Abstract:**

**Figure:**
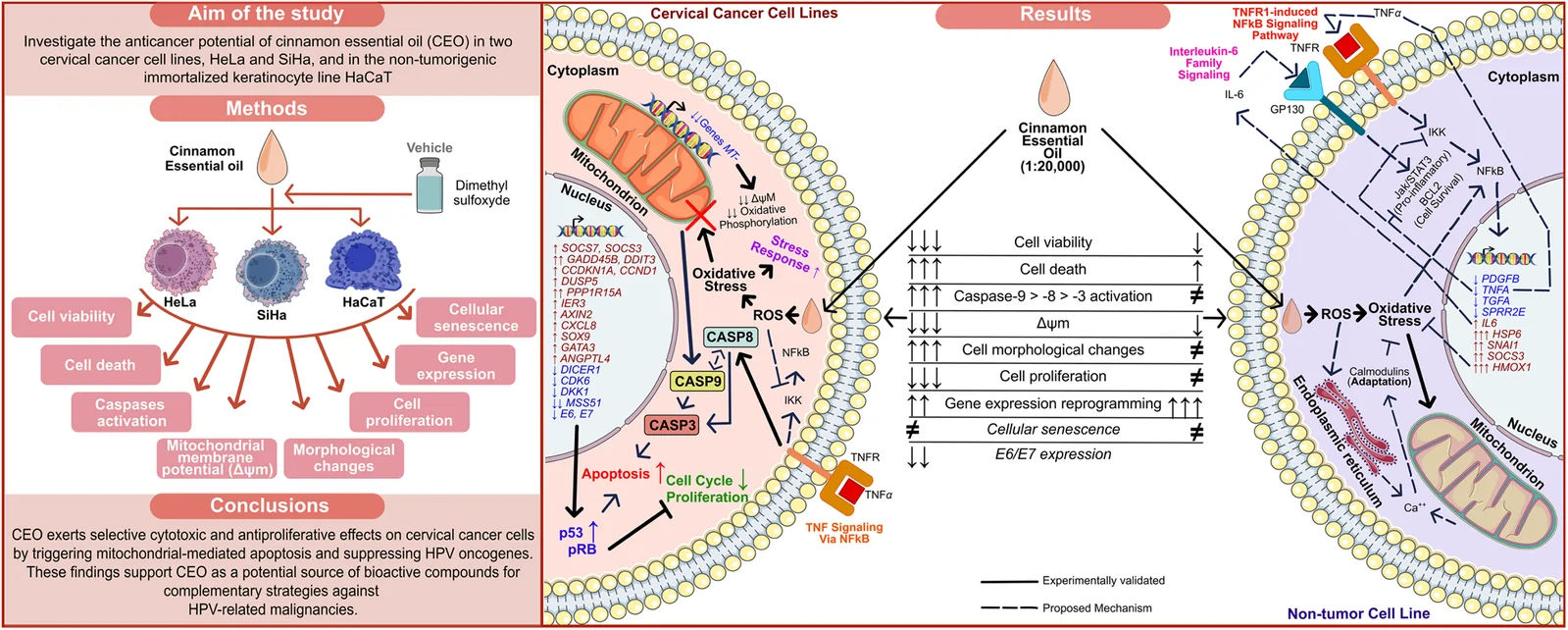

**Supplementary Information:**

The online version contains supplementary material available at https://doi.org/10.1186/s12906-026-05471-7.

## Highlights

- CEO shows dose-dependent and selective killing of cervical cancer cells.
- CEO reduces proliferation without inducing senescence.
- CEO triggers apoptosis via mitochondrial depolarization and caspase activation.
- HPV E6 and E7 oncogenes are downregulated by CEO in tumor cells.
- CEO modulates p53, TNFα/NF-κB, and mTORC1 pathways in cancer lines.

**Supplementary Information:**

The online version contains supplementary material available at https://doi.org/10.1186/s12906-026-05471-7.

## Background

Cervical cancer remains a major public health issue, with an estimated 662,301 new cases and 348,874 deaths worldwide in 2022. It is the fourth most common malignancy and cause of cancer death among women globally, following breast, colorectal, and lung cancers [1–3]. Although the disease is largely preventable, nearly 90% of deaths occur in low- and middle-income countries, reflecting stark global inequalities [4]. Persistent infection with high-risk human papillomavirus (HPV) types, particularly HPV-16 and HPV-18, is the principal etiological factor driving cervical carcinogenesis [5–9]. The viral oncoproteins E6 and E7 play pivotal roles in this process by inactivating the key tumor suppressors p53 and pRb, thereby promoting uncontrolled cell proliferation, evasion of apoptosis, and genomic instability [10–14]. Despite advances in screening, vaccination, and therapeutic strategies, treatment options for established cervical cancer remain limited. Furthermore, current chemotherapeutic regimens often result in cytotoxic side effects and the emergence of drug resistance [15–17]. Therefore, there is an urgent need to identify novel, effective, and safer therapeutic alternatives.

In recent years, the World Health Organization (WHO) has emphasized the importance of exploring bioactive compounds derived from natural or plant-based sources as part of its global strategy to develop innovative, accessible, and sustainable therapeutic agents [18]. Natural products have historically been a cornerstone in drug discovery, providing structurally diverse molecules with potent biological activities [19–21]. In this context, essential oils and their active constituents have attracted attention due to their multifaceted pharmacological effects, including antioxidant, anti-inflammatory, antimicrobial, and anticancer properties [22–27]. Investigating their molecular mechanisms of action may contribute to the discovery of new agents that complement or enhance conventional cancer therapies.

Among these, cinnamon essential oil (CEO), extracted from *Cinnamomum zeylanicum*, has demonstrated a broad spectrum of biological activities, including antioxidant, antimicrobial, and antitumour effects in various cancer models [28–32]. Its main bioactive component, cinnamaldehyde, has been reported to modulate key cellular processes such as oxidative stress, apoptosis, and mitochondrial function [33–41]. Despite growing evidence of CEO’s biological activity, comprehensive insights into its molecular mechanisms in cervical cancer are still limited. Notably, there are no comprehensive transcriptomic studies addressing how CEO modulates gene expression, signalling pathways, or viral oncogene activity in cervical cancer models.

In this study, we aimed to investigate the anticancer potential of CEO in two cervical cancer cell lines, HeLa and SiHa, and in the nontumorigenic immortalized keratinocyte line HaCaT. We evaluated CEO’s ability to reduce cell viability, induce apoptosis, and elicit characteristic morphological changes associated with programmed cell death. In addition, caspase activation, mitochondrial membrane potential alterations, proliferation, and cellular senescence were assessed to delineate its cytotoxic mechanisms. To further explore its molecular impact, we conducted next-generation RNA sequencing (RNA-seq) followed by transcriptomic and pathway enrichment analyses, alongside quantitative PCR (qPCR) validation of key genes and protein–protein interaction analyses. Importantly, we also examined CEO’s effect on the expression of HPV *E6* and *E7* oncogenes. Together, these findings will provide new insights into the cellular and molecular responses elicited by CEO and support its potential as a promising natural compound with therapeutic relevance in cervical cancer.

## Methods

### Cells and cell culture

All cervical cancer cell lines, as well as the nontumorigenic human epithelial cell line HaCaT, were obtained from the German Cancer Research Center (DKFZ, Heidelberg, Germany) and authenticated using multiplex human cell line authentication tests conducted by Multiplexion GmbH (Friedrichshafen, Germany). Cells were cryopreserved in liquid nitrogen until they were required for experiments. For use, cells were quickly thawed in a 37 °C water bath and centrifuged at 1,000 rpm for 10 min to remove residual dimethyl sulfoxide from the freezing process. The resulting cell pellet was then resuspended in fresh Dulbecco’s modified Eagle’s medium (DMEM) supplemented with L-glutamine (584 mg/L), sodium pyruvate (110 mg/L), D-glucose (1 g/L) (Cat. No. 10567–014 Gibco, Thermo Fisher Scientific, Waltham, MA, USA), 10% fetal bovine serum (FBS) (Cat. No. 35–010-CV, Corning, NY, USA), penicillin (100 U/mL), streptomycin (100 µg/mL) and amphotericin (0.25 µg/mL) (Cat. No. 15240–062 Gibco, Thermo Fisher Scientific, Waltham, MA, USA). Cell cultures were maintained at 37 °C in a humidified atmosphere with 5% CO₂ and grown until reaching 80–90% confluence. Once confluence was achieved, the cells were detached with 0.25% trypsin–EDTA (Cat. No. 25–053-CI Corning, Manassas, VA, USA) for subsequent experiments.

### Reagents

The cinnamon essential oil (CEO), Cinnamon® (*Cinnamomum zeylanicum*, Lot No. 30030608), was obtained from dōTERRA® International (Pleasant Grove, UT, USA). According to the manufacturer's certificate of analysis, the major bioactive constituents include cinnamaldehyde (50–70%), cinnamyl acetate (2–15%), and eugenol (1–10%). The essential oil was diluted at a 1:2 ratio in dimethyl sulfoxide (DMSO; Cat No. 67–68-5 Sigma‒Aldrich, MA, USA) to prepare a stock solution (CEO-DMSO).

### Treatments preparation

Treatment dilutions were prepared to the following final concentrations by diluting the CEO-DMSO stock solution in fresh supplemented DMEM: 1:40,000 (26.75 µg/mL), 1:20,000 (53.5 µg/mL), 1:15,000 (71.33 µg/mL), 1:12,500 (85.6 µg/mL), 1:10,000 (107.0 µg/mL), 1:5,000 (214.0 µg/mL), 1:1,000 (1,070.0 µg/mL), and 1:500 (2,140.0 µg/mL). These concentrations were calculated based on the density of the essential oil (approximately 1.07 mg/µL).

### Cell viability assay

Cells were seeded in 96-well, flat-bottom, tissue-culture-grade microplates (Cat: 83.3924 Sarstedt, Nümbrecht, Germany) at densities of 2.5 × 10^4^ for HeLa, 3 × 10^4^ for SiHa, and 4 × 10^4^ for HaCaT cells per well. The plates were incubated at 37 °C in a humidified atmosphere with 5% CO_2_. Treatment dilutions were prepared at the following concentrations: 1:40,000, 1:20,000, 1:15,000, 1:12,500, 1:10,000, 1:5,000, 1:1,000, and 1:500 using the CEO-DMSO stock solution in fresh supplemented DMEM (as described in the cell culture section). A 1:500 dilution of DMSO in supplemented DMEM was used as the vehicle control. Treatments were performed in sextuplicate at 100 μL per well, including untreated and vehicle-only controls. The plates were incubated for 24 h at 37 °C in 5% CO_2_. After treatment, 10 μL of WST-1 cell proliferation reagent (Cat. No. 05–015–944–001, Roche Diagnostics, Mannheim, Germany) was added to each well. Absorbance was measured at 480 nm with correction at 600 nm after 2 and 3 h using a Sinergy HT multimode microplate reader (Cat. No. 7091000, BioTek Instruments, Inc., Winooski, VT). The 50% inhibitory concentration (IC₅₀) was calculated using the formula previously used by Piña-Cruz et al*.* [42], which was originally described by Sebaugh.

### Cell death determination

Cells were seeded in 12-well plates (Cat. No. 83.3921 Sarstedt, Nümbrecht, Germany) at densities of 2.5 × 10^5^ for HeLa cells, 3 × 10^5^ for SiHa cells, and 4 × 10^5^ for HaCaT cells per well and incubated overnight in supplemented DMEM. The cells were then treated with CEO at dilutions of 1:40,000, 1:20,000, 1:15,000, 1:12,500, 1:10,000, 1:5,000, 1:1,000, or 1:1,000 DMSO solution as a vehicle control for 24 h. After treatment, the cells were detached with trypsin and stained using the FITC Annexin V Apoptosis Detection Kit with propidium iodide (PI) protocol (Cat. No. 640914, BioLegend, CA, USA) according to the manufacturer’s instructions. Samples were analysed by flow cytometry (Beckman Coulter Cytoflex), acquiring a minimum of 10,000 events per sample. Cells positive for PI, annexin V (AV), or both (PI⁺/AV⁺) were considered dead, while double-negative (PI⁻/AV⁻) cells were considered viable.

### Visualization of microscopic cellular damage

Cells were seeded in 12-well plates (Sarstedt, Cat: 83.3921, Nümbrecht, Germany) at a density of 2.5 × 10^5^ for HeLa, 3 × 10^5^ for SiHa, and 4 × 10^5^ for HaCaT per well. CEO dilutions of 1:20,000, 1:15,000, 1:12,500, 1:10,000, and 1:5,000 were prepared in supplemented DMEM, along with a 1:5,000 DMSO vehicle control. After 24 h of incubation, photomicrographs were acquired using a ZEISS Primovert inverted cell culture microscope equipped with an AxioCam ERc 5 s camera using a 40 × objective (Carl Zeiss, Germany). Images were captured and processed using ZEISS ZEN software (v. 3.8.1).

### Cell proliferation (EdU incorporation assay)

Cells were seeded in 12-well plates at densities of 2 × 10^5^ (HeLa), 2.5 × 10^5^ (SiHa), and 4 × 10^5^ (HaCaT) cells per well. Following overnight incubation, the cells were treated for 24 h with DMSO, CEO (1:20,000), or doxorubicin (1 µM). This time point, selected based on preliminary time-course experiments and IC₅₀ values from the WST-1 assay, corresponds to a stage at which CEO-induced cytotoxicity was already apparent, allowing for the capture of early antiproliferative responses before extensive cell death occurred and thus enabling a clearer assessment of DNA synthesis inhibition. A uniform 24-h exposure was applied across all cell lines to allow direct comparisons, regardless of differences in their doubling times. After treatment, the cells were incubated with 10 mM EdU for 4 h at 37 °C and 5% CO_2_ and then fixed and permeabilized using the EdU Cell Proliferation Kit with iFluor 647 Azide (Cat. No. ab222421 Abcam Cambridge, MA, USA). Fluorescence was analysed by a Cytek Aurora spectral flow cytometer (Cytek Biosciences, Fremont, CA, USA). Data acquisition was performed using SpectroFlo™ software (v.3.0.1), and analysis was conducted with FlowJo™ software (v.10.9.0, BD Biosciences Ashland, OR, USA). This cytometer and software were used in the subsequent flow cytometry assays.

### Cell viability (Sulforhodamine B Assay)

Cells were plated in 96-well plates at the following densities: 5 × 10^3^ for HeLa, 6.25 × 10^3^ for SiHa, and 1 × 10^4^ for HaCaT. After 24 h of incubation, cells were treated with CEO dilutions (1:40,000, 1:30,000, 1:20,000, and 1:10,000), DMSO (vehicle control), and doxorubicin (0.5 μM and 1 μM) as a positive control for 72 h. Without removing the medium, 50 µL of fixative was added to each well, and the plates were incubated at 4 °C for 1 h. The wells were then washed three times with distilled water, stained with 45 µL of sulforhodamine B (Cat. No. ab235935 Abcam Cambridge, MA, USA) for 15 min, washed four times, and air-dried. Microscopy images were captured with a ZEISS Primovert inverted cell culture microscope using a 40 × objective. Bound dye was solubilized in 200 µL of 10 mM Tris base (pH 10.5), which is the standard buffer recommended for this step. The plates were gently shaken for 10 min at room temperature, and the absorbance was measured at 565 nm using a Synergy HT microplate reader.

### Caspase-3, −8, and −9 activation

HeLa, SiHa, and HaCaT cells were seeded in 12-well plates at densities of 2 × 10^5^, 2.5 × 10^5^, and 3 × 10^5^ cells per mL, respectively, in 1 mL of supplemented DMEM. After 24 h, the cells were treated with CEO (1:20,000 dilution) for an additional 24 h at 37 °C with 5% CO₂. Following treatment, cells were harvested, washed once with PBS, detached with trypsin–EDTA and stained with caspase 3, 8 or 9 activity assay kits (Abcam, Cat. Nos. ab65613, ab65614, ab65615, respectively), according to the manufacturer’s protocols. Stained samples were analysed by spectral flow cytometry. Mean fluorescence intensity (MFI) values were obtained using FlowJo software (v.10.9.0, BD Biosciences Ashland, OR, USA) and plotted in GraphPad Prism (v.8.0.2, San Diego, CA, USA).

### Mitochondrial membrane potential

HeLa (2 × 10^5^), SiHa (2.5 × 10^5^), and HaCaT (4 × 10^5^) cells were seeded in 12-well plates with 1 mL of DMEM and incubated overnight. Afterwards, the cells were treated with DMSO or CEO (1:20,000) for 24 h. After treatment, the cells were harvested, washed once with PBS, detached with trypsin–EDTA, transferred to labelled flow cytometry tubes, and centrifuged at 1,000 rpm for 10 min. The resulting cell pellets were resuspended in 500 µL of staining solution (prepared by diluting 1 µL of JC-10 dye in 5 mL of assay buffer (Cat. No. ab112133 Abcam, Cambridge, MA, USA)) and incubated for 30 min at 37 °C with 5% CO₂. Samples were analysed immediately by spectral flow cytometry. The red/green fluorescence ratio (570/520 nm) was calculated based on the MFI values and plotted using GraphPad Prism (v.8.0.2, GraphPad Software, San Diego, CA, USA).

### Induction and assessment of cellular senescence

Cells were seeded in 12-well plates at 1 × 10^5^ (HeLa), 1.2 × 10^5^ (SiHa), and 1.6 × 10^5^ (HaCaT) cells per well in 1 mL of medium. After overnight incubation, the cells were treated with DMSO, CEO (1:20,000), or doxorubicin (2 μM) for 48 h. Senescence was assessed using the Senescence Detection Kit for Flow Cytometry (Cat. No. ab228562 Abcam, Cambridge, MA, USA). Following treatment, the cells were stained for 1 h at 37 °C according to the manufacturer´s instructions, washed twice, and resuspended in 300 µL of assay buffer for immediate analysis by spectral flow cytometry. Data were analysed using FlowJo™ software (v.10.9.0, BD Biosciences, Ashland, OR, USA).

### RNA extraction

Cells were seeded in 100 mm tissue culture dishes (Cat. No. 430167 Corning, NY, USA) at densities of 2.5 × 10^6^ for HeLa, 3 × 10^6^ for SiHa, and 4 × 10^6^ for HaCaT cells. After overnight incubation, the cells were treated with a 1:20,000 dilution of CEO or DMSO (vehicle control) for 4 h. Total RNA was isolated using the Quick-RNA MiniPrep kit (Cat. No. R1055, Zymo Research Corp., Irvine, CA, USA) following the manufacturer's instructions. The RNA concentration was determined by measuring absorbance at 260/280 nm using the Synergy HT microplate reader and stored at −80 °C until use.

### RNA-Seq analysis

RNA integrity was assessed with a Bioanalyzer 2100 (Agilent Technologies, Santa Clara, CA, USA) using the Agilent RNA 6000 Nano Kit (Cat. No. 6067–1511 Agilent Technologies, Santa Clara, CA, USA). Only samples with RNA integrity numbers (RIN) > 7 were used for sequencing. A total of 3.0 µg of RNA per sample was submitted to Novogene Corporation Inc. (Sacramento, CA, USA) for next-generation sequencing using NovaSeq 6000 technology (Illumina, San Diego, CA, USA). The experiment included two biological replicates per condition (CEO-treated and DMSO vehicle control) for each cell line (HeLa, SiHa, and HaCaT). The raw sequencing data have been deposited in the GEO Data repository under the accession number GSE311189.

### Bioinformatics

Raw reads were analysed using R (v. 4.3.1), RStudio (v. 2023.09.1 + 494), and the Galaxy platform (https://www.usegalaxy.org, accessed August 2024). Quality was assessed with the FastQC tool (Galaxy, V. 0.74 + galaxy0). Reads were mapped and aligned to the Human Reference Genome GRCh38.p14v46 using the align function from the Rsubread package (Ver. 2.14.2). The resulting BAM files were used to generate read count tables with the FeatureCounts function. Differentially expressed genes (DEGs) were identified with DESeq2 (v. 1.42. 0) R package. The enrichment analysis was performed using the Kyoto Encyclopedia of Genes and Genomes (KEGG) database in R using the PathFindeR package (v. 2.3.1) and hallmark gene data sets using Gene Set Enrichment Analysis (GSEA) software (v. 4.3.2) [43].

### Protein–Protein Interaction (PPI) network analysis

To explore interactions among proteins encoded by the differentially expressed genes (DEGs) identified through RNA-seq, only genes meeting the criteria of adjusted p value (*padj)* < 0.05 and |log₂ fold change (FC)|≥ 2.0 were selected. These DEGs were uploaded to the STRING database (https://string-db.org/) using the "Multiple proteins" option with *Homo sapiens* set as the reference organism. A minimum interaction confidence score of 0.4 ~ 0.7 was applied. Unconnected proteins were filtered out to improve clarity and focus on biologically relevant interactions.

### Quantitative Polymerase Chain Reaction (qPCR)

Total RNA, isolated as previously described, was used for quantitative polymerase chain reaction (qPCR). Complementary deoxyribonucleic acid (cDNA) synthesis was performed using the Transcriptor First Strand cDNA Synthesis Kit (Cat. No. 04 379 012 001, Roche Diagnostics, Basel, Switzerland). qPCR was carried out on the LightCycler 2.0 platform (Roche Diagnostics, Basel, Switzerland) using the LightCycler FastStart DNA Master plus SYBR Green I Kit (Cat. No. 03515869001, Roche Diagnostics, Basel, Switzerland). Primers were designed using the NCBI Primer-BLAST tool (https://www.ncbi.nlm.nih.gov/tools/primer-blast/). The expression data were normalized using *RPS18*, *RPLP0, and GAPDH* as reference genes. Primer sets for the target genes (*HSPA6*, *GADD45B*, *PPP1R15A*, *HMOX1*, *ATF3*, *DDIT3*, *CDKN1A*, *MT-CO1*, *MT-CO2*, *MTCO2P12*, *MT-CO3*, *CCND1*, *JUN*, *EGR1*, *PGF*, *KLHL24*, *SNAI1*, *SOCS3*, *PMAIP1*, *E6* HPV-16, *E6* HPV-18, *E7* HPV16, *E7* HPV18) are summarized in Table 1. The results were analysed using LightCycler Software v. 4.1, and relative gene expression was calculated using the 2⁻ΔΔCp method and expressed as log₂ fold change.

**Table 1:** Primer sequences and qPCR conditions for target gene expression analysis. The table includes the forward (F-) and reverse (R-) primer sequences, amplicon length, and annealing temperature for each target gene

Gene	Sequence (5’−3’)	Amplicon length (pb)	Annealing temp (°C)
*ATF3*	F-TCT GCG CTG GAA TCA GTC R-CTC CTT CTT CTT GTT TCG GCA C	150	59
*CCND1*	F-CGC ACG ATT TCA TTG AAC AC R-CAG CAG GGC TTC GAT CTG	296	60
*CDKN1A*	F-TGT ACC CTT GTG CCT CGC T R-GCT TCC TGT GGG CGG ATT A	163	64
*DDIT3*	F-TTC AGA TGA AAA TGG GGG TAC R-ACC ACT CTG TTT CCG TTT CC	238	62
*E*6 HPV-16	F-GAG ACA TTT TAT GCA CCA AA R-CTC CAT GCA TGA TTA CAG C	499	60
*E6* HPV-18	F-AAT ACT ATG GCG CGC TTT GA R-TTG CCT TAG GTC CAT GCA TAC T	510	60
*E7* HPV-16	F-TAG AGA AAC CCA GCT GTA ATC A R-AGG ATC AGC CAT GGT AGA TTA T	336	60
*E7* HPV-18	F-CGC AGA GAA ACA CAA GTA TAA T R-GAT CAG CCA TTG TTG CTT A	363	60
*EGR1*	F- GAA CGC AAG CAT ACC A R- GGT TGC TGT CAT GTC CGA AAG	375	60
*GADD45B*	F-CAT CAA CAT CGT GCG GGT GT R-GTA GGG GAC CCA CTG GTT GT	205	66
*GAPDH*	F-CAC TGC CAC CCA GAA GAC TGT G R-TGT AGG CCA TGA GGT CCA CCA C	449	56
*HMOX1*	F-CTG CTG ACC CAT GAC ACC AA R-CAA CTG TCG CCA CCA GAA AG	187	61
*HSPA6*	F-CCA GAG GAA CGC CAC TAT CC R-GGA GGG ATG CCA CTG AGT TC	156	62
*JUN*	F- TGG AAA GTA CTC CCC TAA CCT R- CTG AAA CAT CGC ACT ATC CTT	250	58
*KLHL24*	F- GAA AGG GAA AGG AGA AGC AGG A R- GAG GAC AGC CAT TTT GTG ACG	263	62
*MT-CO1*	F-CGA CGT TAC TCG GAC TAC CC R-GAA TGT GTG GTA GGG TGG GG	204	62
*MT-CO2*	F-GTA CTC CCG ATT GAA GCC CC R-CGA TGG GCA TGA AAC TGT GG	202	62
*MTCO2P12*	F-GGT TGA AGC CCC CAT TCG TA R-TTC AGA GCA TTG GCC GTA GT	160	61
*MT-CO3*	F-CGG CAT CTA CGG CTC AAC AT R-AAT GCC AGT ATC AGG CGG C	161	61
*PDF*	F- AAG GGG GCC CTG CTA CCT GT R- CCC CAG GCA ACC ACT GTT CTC	218	62
*PMAIP1*	F-GAG ATG CCT GGG AAA AAG G R-TCC TGA GCA GAA GAG TTT GGA	313	58
*PPP1R15A*	F-TTC CGA GTG GCC ATC TAT GT R-ATG GAC AGT GAC CTT CTC GG	177	59
*RPLP0*	F-CCT CAT ATC CGG GGG AAT GTG R-GCA GCA GCT GGC ACC TTA TTG	95	58
*RPS18*	F-CGA TGG GCG GCG GAA AA R-CAG TCG CTC CAG GTC TTC ACG G	283	60
*SNAI1*	F- CGA AAG GCC TTC AAC TGC AA R- CGC CTG GCA CTG GTA CTT CTT	270	60
*SOCS3*	F- CAA GGA CGG AGA CTT CGA TTC R- GGT CAC TGC GCT CCA GTA GAA	273	60

## Results

### Cinnamon essential oil induces cell death and reduces viability in a dose-dependent manner in cervical cancer cell lines

To evaluate the cytotoxic effects of CEO on cervical cancer cell lines (HeLa and SiHa) and nontumorigenic keratinocytes (HaCaT), we assessed cell viability using the WST-1 assay and cytotoxicity by Annexin V/Propidium Iodide (AV/PI). Cells were treated with a range of dilutions of CEO from 1:40,000 to 1:500. DMSO was also included as vehicle-treated cells and showed no significant differences compared to untreated cells (Control).

**Fig. 1:**
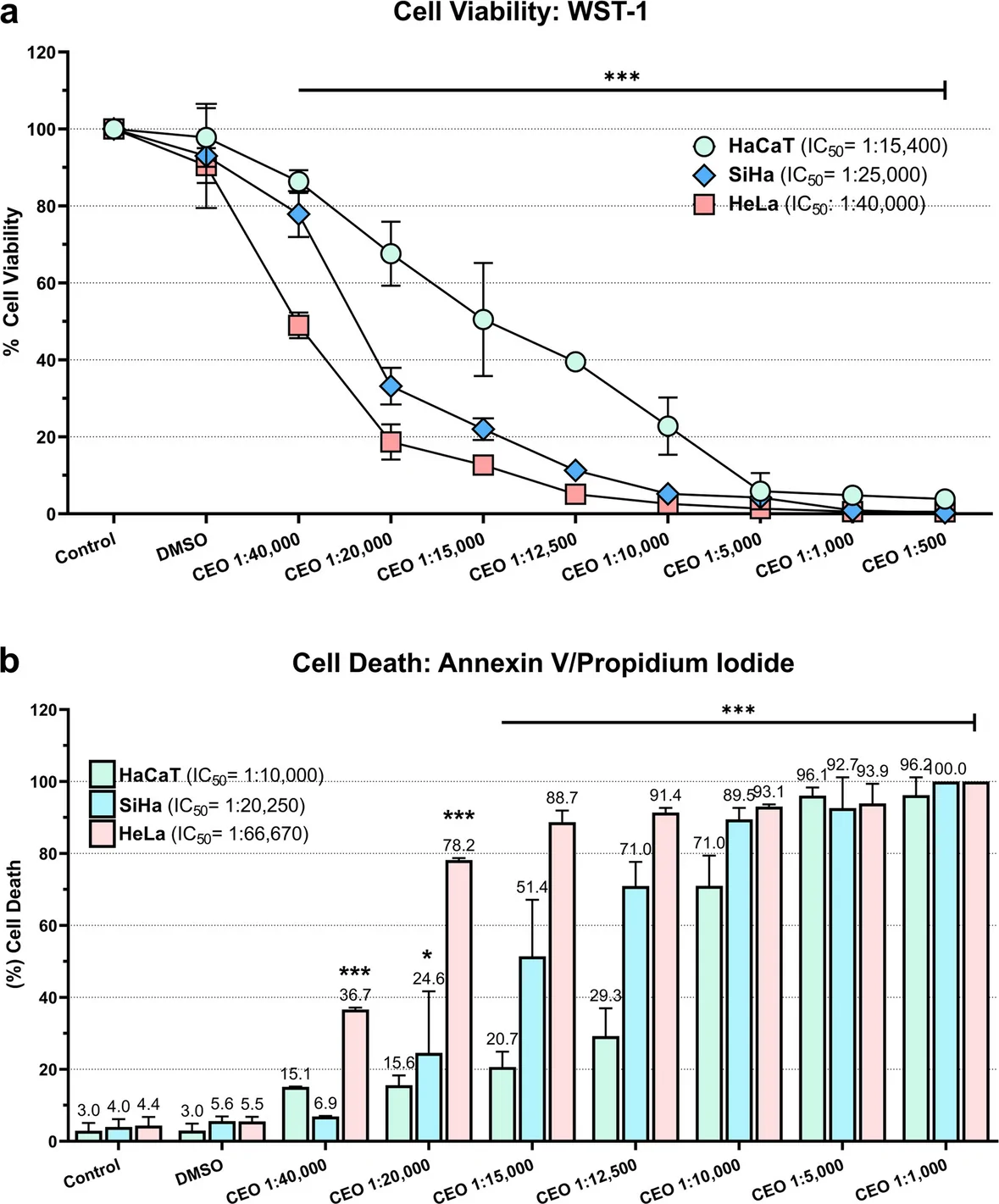
Cytotoxic effect of cinnamon essential oil (CEO) on cervical cancer cells and nontumorigenic keratinocytes. **a** WST-1 viability assay showing the percentage of viable cells after 24 h of treatment with increasing CEO concentrations. Untreated cells (control) and cells treated only with vehicle (DMSO) were included. The half-maximal inhibitory concentration (IC_50_) values are indicated for each cell line. In addition, cell death was quantified in parallel by (**b**) Annexin V/Propidium Iodide (PI) staining performed by cytometry. Annexin V–positive, PI–positive, and double-positive populations were collectively considered dead cells. Data are presented as the mean ± SD from three independent experiments. Statistical significance is indicated as ***p < 0.001 vs. control

As shown in Fig. 1a, CEO exhibited a dose-dependent effect on cell viability (WST-1 assay), with IC_50_ values of 1:40,000 for HeLa, 1:25,000 for SiHa, and 1:15,400 for HaCaT cells, indicating that HaCaT cells were the most resistant. The most pronounced difference occurred at the 1:20,000 dilution, and CEO reduced cell viability to 20.8% in HeLa, 33.2% in SiHa, and 83.2% in HaCaT (*p* < 0.05).

CEO-induced cytotoxicity was confirmed to be due to cell death, as shown by Annexin V/Propidium Iodide (AV/PI) staining (Fig. 1b), showing massive cell death (78.2%) in HeLa cells and 24.6% in SiHa cells at the same dilution versus only 15.6% in HaCaT cells. The IC_50_ values derived from the AV/PI assay were 1:66,670 for HeLa, 1:20,250 for SiHa, and 1:10,000 for HaCaT, reflecting the greater sensitivity of the apoptosis assay compared to the metabolic activity assay. Based on these results, a 1:20,000 dilution was selected for subsequent experiments.

### Morphological changes and EdU incorporation assays validate the antiproliferative effect of cinnamon essential oil

**Fig. 2:**
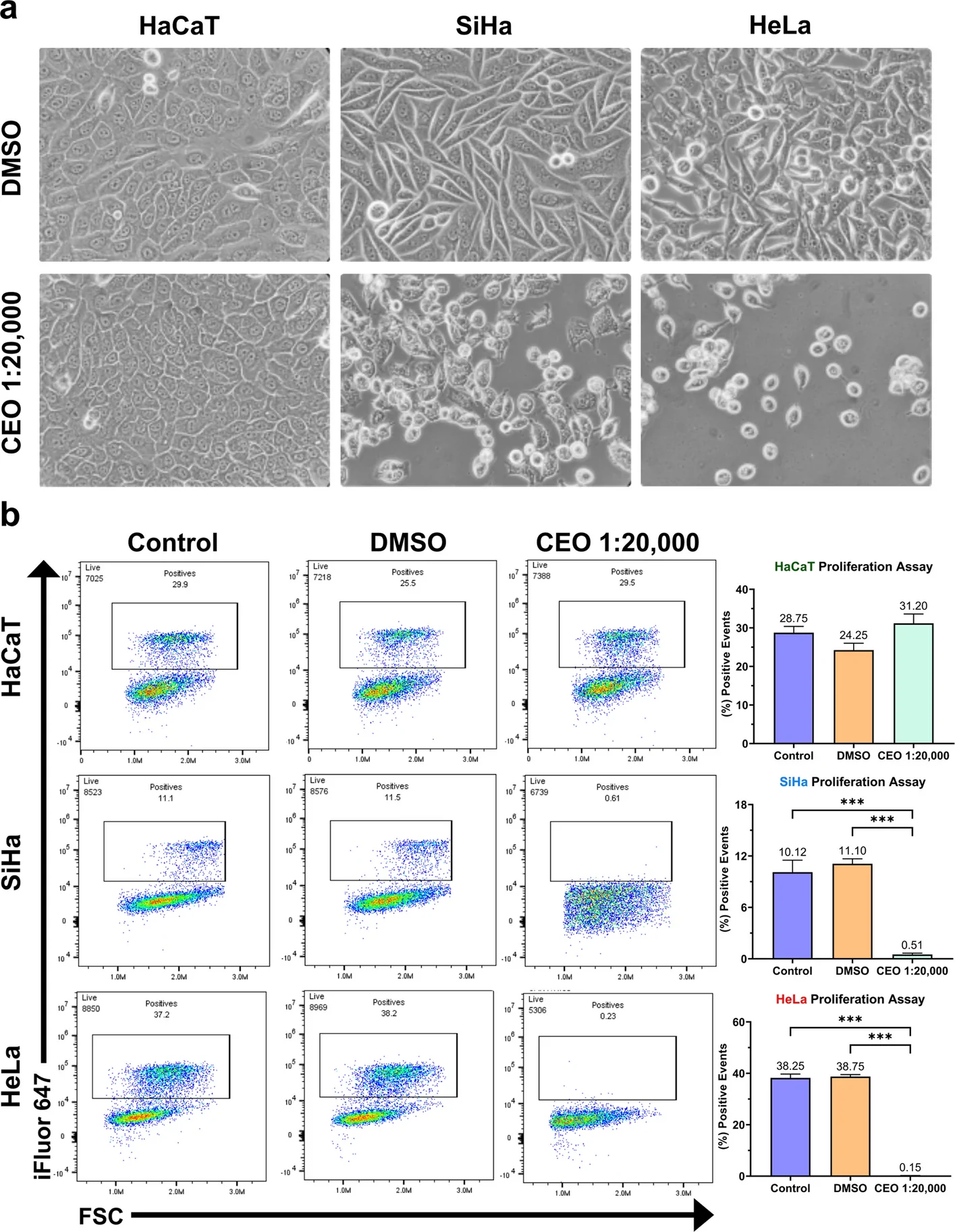
Morphological changes and cell proliferation assay after 24 h of incubation with CEO. **a** Phase-contrast microscopy images (40 × magnification) depict the morphological alterations in HaCaT, SiHa, and HeLa cells treated with DMSO or CEO (1:20,000 dilution). **b** Cell proliferation assay measured by EdU incorporation, detected with iFluor 647, and analysed by flow cytometry. Representative dot plots display forward scatter (FSC-A) versus EdU–iFluor 647 fluorescence intensity. The gated region indicates EdU-positive cells actively undergoing DNA synthesis. On the right side of the figure, bar graphs summarize the percentage of EdU-positive cells for each condition across the four cell lines. Statistical significance is indicated as ***p < 0.001 compared to the control group

To evaluate the effect of cinnamon essential oil (CEO) on cell morphology, CC-derived cell lines and nontumorigenic HaCaT keratinocytes were exposed to a 1:20,000 dilution for 24 h. As shown in Fig. 2a, phase-contrast microscopy revealed marked morphological alterations in CEO-CC-treated cells, including cell rounding, shrinkage, and detachment, consistent with apoptotic death. In contrast, DMSO-treated cells maintained a confluent monolayer with intact cell‒cell junctions and typical epithelial morphology (consistent with untreated controls from preliminary experiments), and HaCaT cells exhibited no significant changes upon CEO treatment.

Based on these cytotoxic effects, we next examined whether CEO also affects cell proliferation using a 5-ethynyl-2′-deoxyuridine (EdU) incorporation assay. As illustrated in Fig. 2b, CEO treatment for 24 h significantly reduced cell proliferation in HeLa and SiHa cells (*p* < 0.001), demonstrating a strong antiproliferative effect. Interestingly, in nontumorigenic HaCaT cells, CEO did not inhibit proliferation and instead induced a slight, nonsignificant increase.

### Cinnamon essential oil selectively and potently reduces cellular biomass in cervical cancer cells

**Fig. 3:**
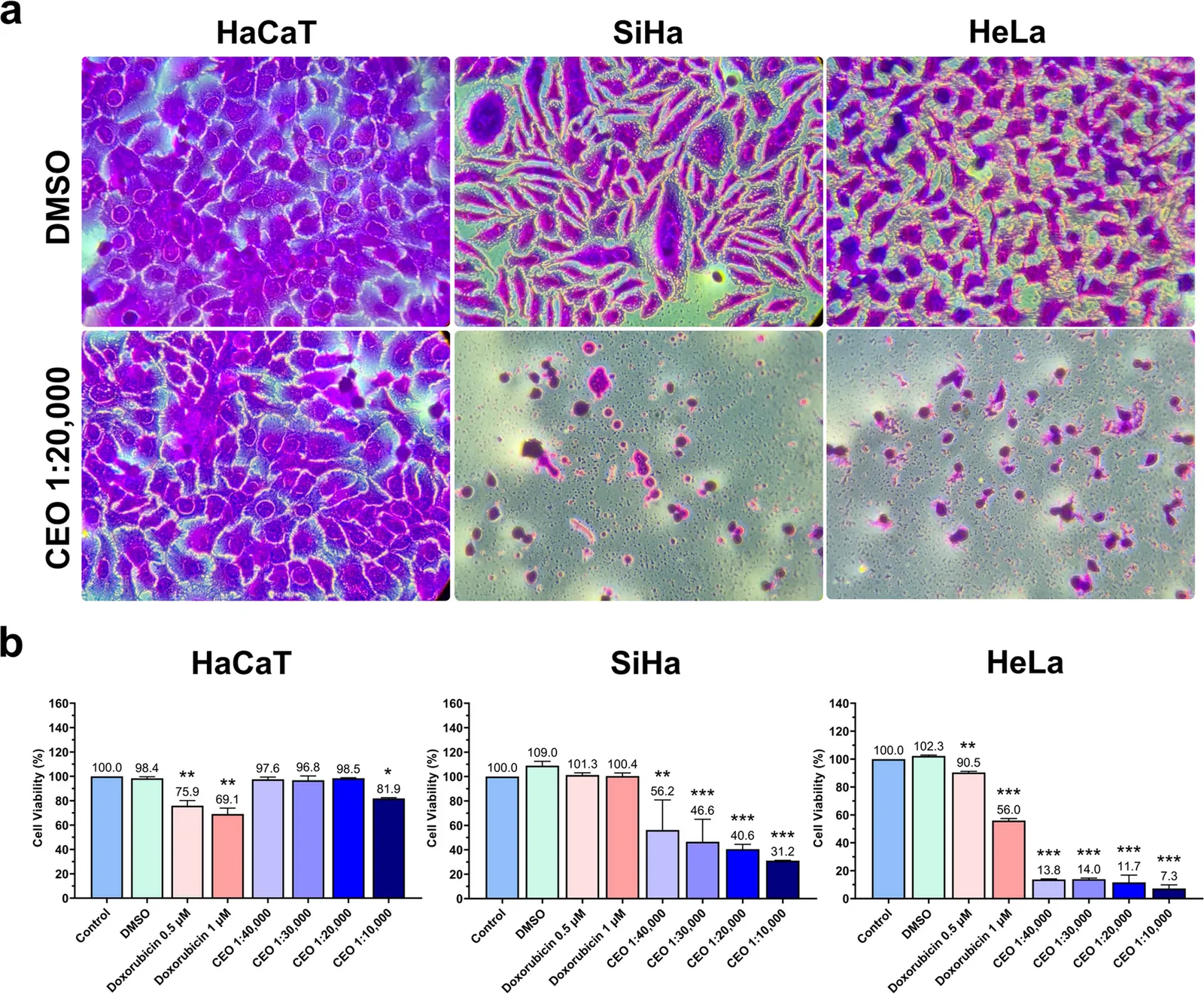
Cell mass reduction observed under phase-contrast microscopy and cell viability assessed by Sulforhodamine B assay. **a** Representative phase-contrast micrographs (40 ×) of HaCaT, SiHa, and HeLa cells after 24 h of treatment with CEO at a 1:20,000 dilution or vehicle control (DMSO). **b** Measurement of total cellular protein content using sulforhodamine B (SRB) staining in HaCaT, SiHa, and HeLa cells. Cells were treated with increasing concentrations (1:40,000–10,000) of CEO, untreated (control), or DMSO (vehicle). Treatments with 0.5 or 1 µM doxorubicin were also included as positive controls. The results are expressed as the percentage of viable cells relative to the untreated control. Statistical significance is indicated as *p < 0.05, **p < 0.01, ***p < 0.001

To further corroborate the antiproliferative effect of CEO, cell viability was assessed using the sulforhodamine B (SRB) assay. Consistent with previous WST-1 and EdU results, microscopic examination revealed that CEO treatment (1:20,000) caused a marked loss of cell density, diminished staining intensity, and morphological features of cytotoxicity, such as shrinkage and detachment (Fig. 3a). In contrast, HaCaT cells retained their morphology and staining intensity. DMSO-treated controls exhibited uniform SRB staining, high cell density, and intact morphology in all cell lines, indicating preserved viability.

Quantitative analysis of SRB absorbance confirmed these morphological observations (Fig. 3b). In HeLa cells, CEO reduced cell viability to below 14% in all tested dilutions (1:40,000 to 1:10,000). In SiHa cells, a clear dose-dependent decrease was observed (56.2% to 31.2% viability) (*p* < 0.001). Conversely, HaCaT viability remained above 96% except at the highest CEO concentration (1:10,000), where it was 81.9%. This confirms the selective cytotoxicity of CEO against malignant cells. Additionally, for comparison, cells were incubated with two concentrations of doxorubicin (0.5 µM and 1 µM). The most affected cells were HeLa (56.0% viability), followed by HaCaT (69.1%); however, SiHa cells, which are generally more resistant to chemotherapeutic agents, were not affected and instead showed slightly accelerated proliferation.

### Cinnamon essential oil triggers apoptosis through caspase- 8 and 9 activation following mitochondrial membrane depolarization.

Given our previous findings that CEO induces cell death, we further investigated the apoptotic mechanisms by assessing the activation of caspase-8 (extrinsic pathway), caspase-9 (intrinsic pathway), and caspase-3 (executioner caspase). Flow cytometry analysis was performed after 24 h of treatment with CEO at a 1:20,000 dilution.

**Fig. 4:**
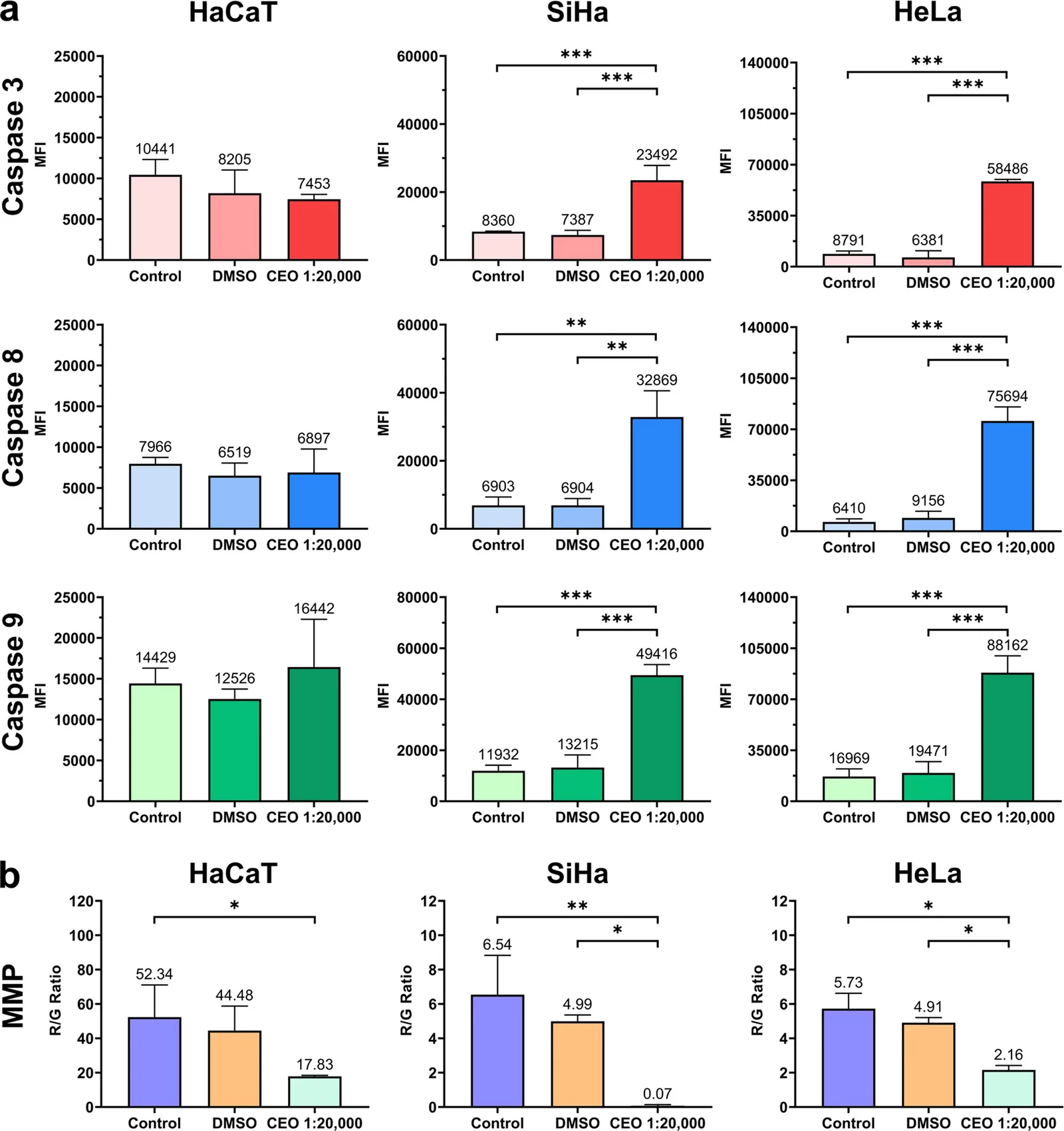
Activation of caspases-3, −8, and −9 and assessment of mitochondrial membrane potential (ΔΨm). **a** Bar graphs represent the mean fluorescence intensity (MFI) of caspase-8 (extrinsic pathway), caspase-9 (intrinsic pathway), and caspase-3 (execution phase) measured by flow cytometry in HaCaT, SiHa, and HeLa cells after 24 h of exposure to CEO (1:20,000 dilution), untreated control, or DMSO (vehicle). Data are expressed as the mean ± SD from at least three independent experiments. **b** Bar graphs show the mitochondrial membrane potential (ΔΨm) assessed using JC-10 dye by flow cytometry. The graph displays the red (viable cells)/green (nonviable or late apoptotic) fluorescence intensity ratio, where a decrease in this ratio indicates a loss of ΔΨm. Statistical significance is indicated as *p < 0.05, **p < 0.01, ***p < 0.001

As shown in Fig. 4a, CEO treatment resulted in a marked and statistically significant increase in the activation of all three caspases in the tumor-derived cell lines HeLa and SiHa compared to both untreated and vehicle-treated controls. In contrast, the nontumorigenic HaCaT cells showed no significant caspase activation, consistent with previous assays demonstrating minimal cytotoxic effects in this cell line. The marked activation of caspase-9 prompted us to examine whether CEO also affects mitochondrial integrity. Therefore, we evaluated changes in mitochondrial membrane potential (Δψm), a key indicator of mitochondrial function and a hallmark of apoptosis.

As shown in Fig. 4b, Δψm was assessed using JC-10 dye after 24 h of CEO treatment. Healthy mitochondria emit red fluorescence, while depolarized mitochondria fluoresce green. CEO treatment caused a significant decrease in the red-to-green fluorescence ratio in HeLa cells from 5.73 (control) and 4.91 (DMSO) to 2.16 (CEO-treated cells; *p* < 0.05), indicating substantial mitochondrial membrane depolarization. SiHa cells showed a more profound drop from 6.54 (control) and 4.99 (DMSO) to just 0.07 in the CEO-treated cells (*p* < 0.001). Interestingly, in HaCaT cells, CEO treatment also induced a measurable reduction in the red/green ratio from 52.34 (control) and 44.48 (DMSO) to 17.83 in CEO (1:20,000 dilution).

### The cytotoxic effect of cinnamon essential oil is mediated primarily by apoptosis rather than senescence

**Fig. 5:**
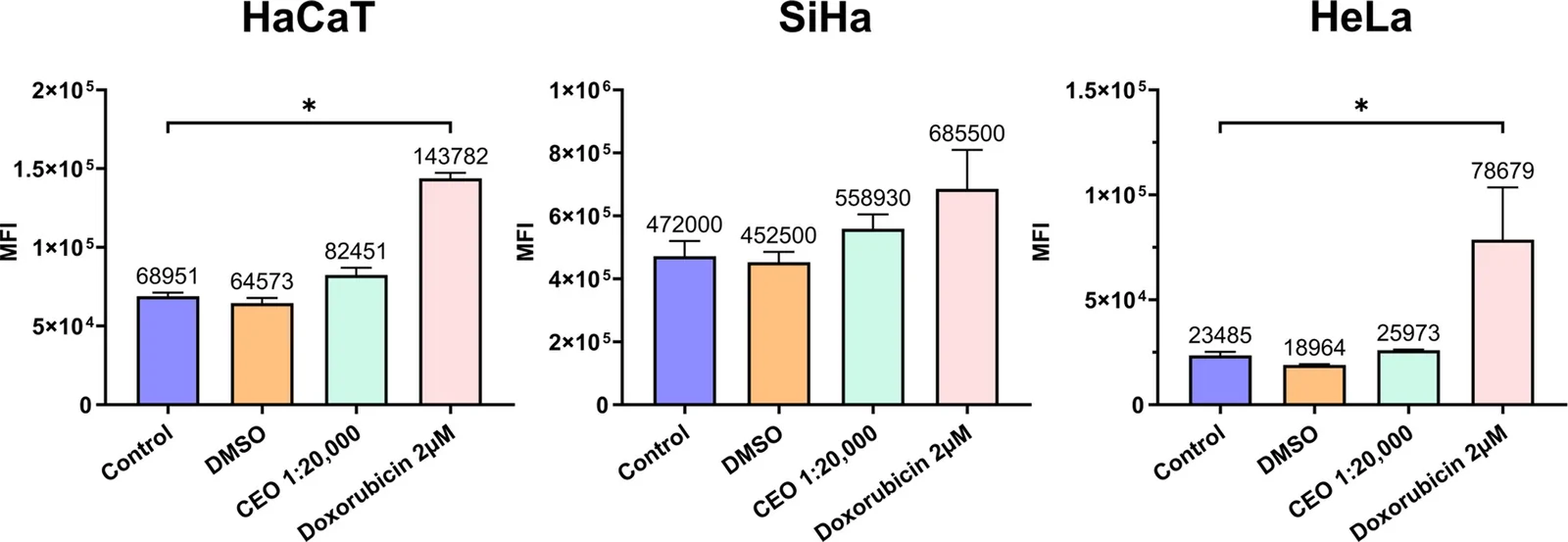
Cellular senescence assessment using β-galactosidase (SA-β-gal) activity by flow cytometry. Bar graph plots depict the mean fluorescent intensity (MFI) of SA-β-gal–positive events in HaCaT, SiHa, and HeLa cells under four experimental conditions: untreated cells (Control), vehicle control (DMSO), cinnamon essential oil treatment (CEO 1:20,000), and a positive control (Doxorubicin) after 48 h of incubation. The results are expressed as the percentage of MFI ± SD from at least three independent experiments. Statistical significance is indicated as *p < 0.05

To evaluate cellular senescence induction, the mean fluorescence intensity (MFI) was measured by flow cytometry after 48 h of treatment with DMSO (vehicle), CEO (1:20,000), or doxorubicin (2 μM, positive control). In HaCaT cells, doxorubicin significantly increased MFI compared to the untreated control (143,782 vs. 68,951; *p* < 0.05), indicating strong senescence induction. In contrast, CEO caused only a modest, nonsignificant increase (MFI: 82,451). In SiHa cells, both CEO (558,930) and doxorubicin (685,500) increased MFI compared to the control (472,000). However, these changes were not statistically significant. Conversely, in HeLa cells, doxorubicin treatment significantly elevated MFI (78,679) compared to the control (23,485; *p* < 0.05), whereas treatment with CEO had no appreciable effect compared to the control or DMSO (25,973 vs. 23,485 or 18,964, respectively). Together, these results demonstrate that while the positive control doxorubicin consistently induced senescence, CEO did not produce a significant or consistent senescent response across cell lines (Fig. 5).

### Cinnamon essential oil downregulates the expression of HPV oncogenes E6 and E7

Having established that CEO induces selective cytotoxicity, we next investigated its impact on the expression of the viral oncogenes *E6* and *E7* from HPV-16 and HPV-18, which are critical drivers of cervical carcinogenesis. Quantitative real-time PCR was performed to assess the mRNA levels of *E6* and *E7* in HPV-18-positive HeLa cells and HPV-16-positive SiHa cells following a 4-h treatment with CEO at a 1:20,000 dilution.

**Fig. 6:**
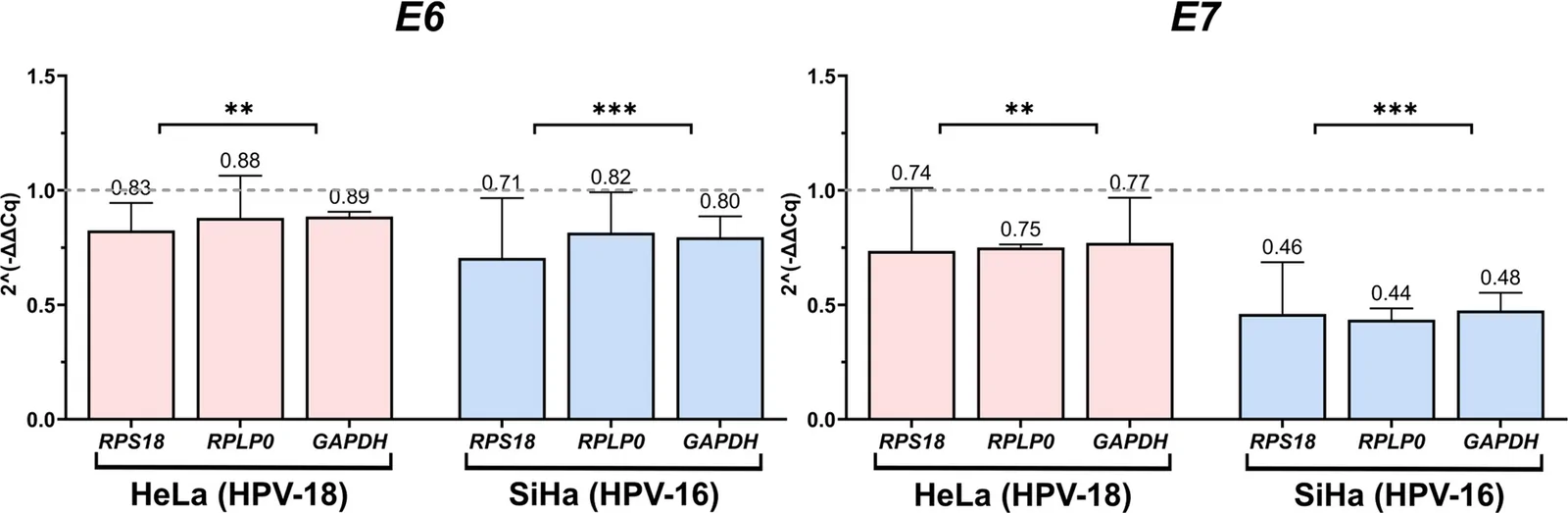
Expression of HPV E6 and E7 oncogenes measured by qPCR. Bar graphs depict the expression levels of *E6* and *E7* in HeLa (red) and SiHa (blue) cells represented as 2^(-ΔΔCq). Normalization was performed using three reference genes (*RPS18*, *RPLP0*, and *GAPDH*). The dashed gray line indicates the expression level of the untreated cells (value set as = 1). Statistical significance is indicated as **p < 0.01, ***p < 0.001

As shown in Fig. 6, CEO treatment resulted in a statistically significant downregulation of both *E6* and *E7* transcripts. In HeLa cells, expression levels were reduced to 0.83-fold for *E6* and 0.75-fold for E7 relative to DMSO controls (*p* < 0.01). A more pronounced downregulation was observed in SiHa cells, where CEO treatment decreased *E6* expression to 0.74 and E7 expression to 0.46 compared to controls (*p* < *0.001*). These findings demonstrate the suppression of key HPV oncogenes, with a stronger inhibitory effect observed in SiHa cells.

### Analysis of differentially expressed genes and enriched pathways following cinnamon essential oil treatment

**Fig. 7:**
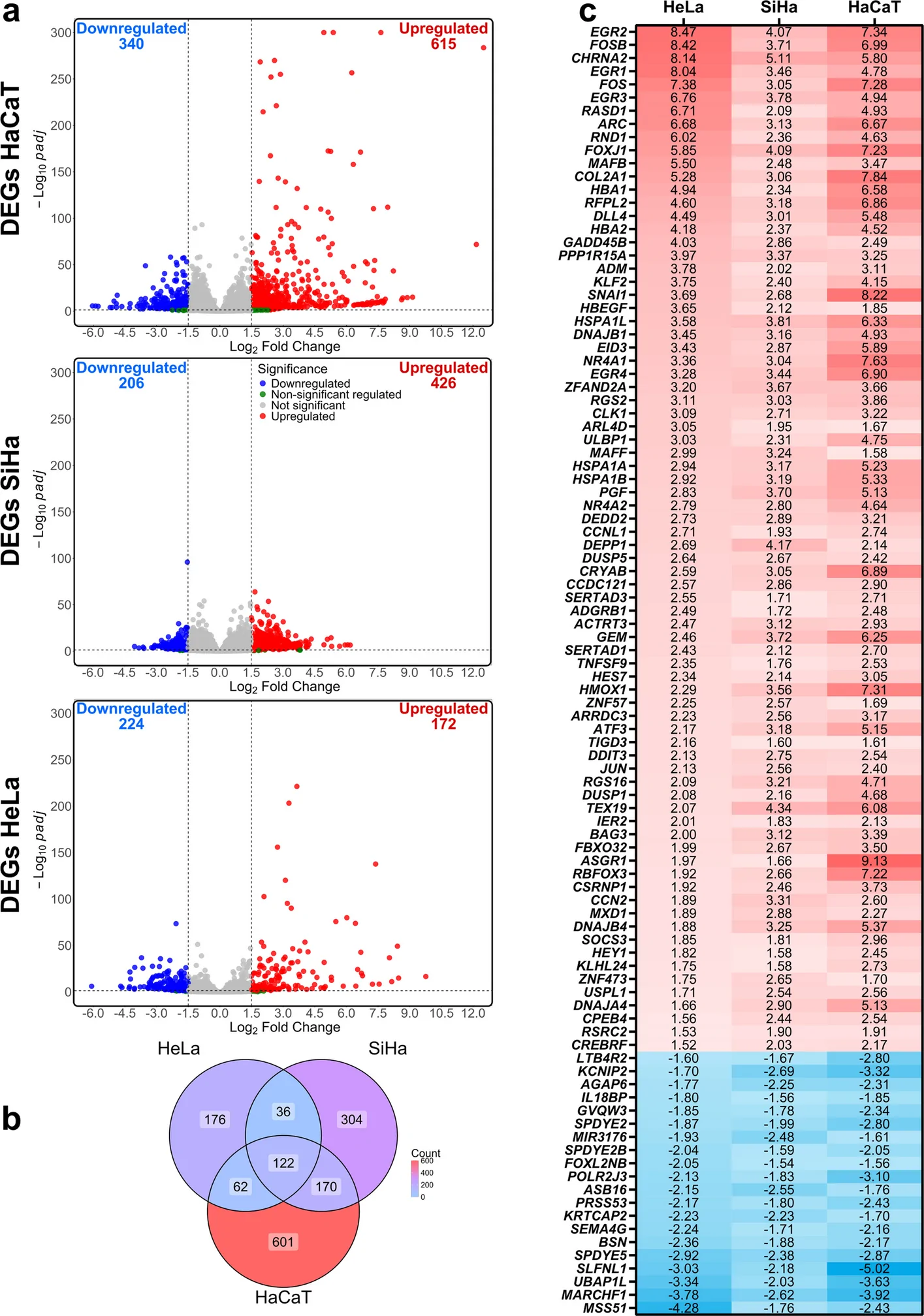
Transcriptomic analysis of CEO-treated cells. **a** Volcano plots showing differentially expressed genes (DEGs) in HaCaT, SiHa, and HeLa cell lines. Upregulated genes are shown in red, downregulated genes in blue, and nonsignificant genes in gray. The total number of DEGs identified in each cell line is indicated below each plot. The x-axis represents the log2-fold change, and the y-axis shows the –log10 adjusted *p* value. Each dot represents a single gene. **b** Venn diagram depicting the unique and shared DEGs among the three cell lines. Color intensity represents the number of DEGs (red = higher number, blue = lower number). **c** Heatmap of the 98 commonly regulated DEGs across all cell lines, displaying log2-fold change values included. Red indicates upregulation, and blue indicates downregulation. These 98 DEGs corresponded to the 122 genes from the Venn diagram after filtering out noncoding genes and novel transcripts. Additional file 1 provides a broader overview of the top differentially expressed genes (both upregulated and downregulated) in each cell line

To elucidate the molecular mechanisms of CEO, we performed transcriptomic profiling on the tumorigenic HeLa and SiHa cell lines and the nontumorigenic HaCaT keratinocytes. CEO exerted a strong modulatory effect on the transcriptome of all three cell lines. The volcano plot (Fig. 7a) illustrates the global transcriptomic landscape in gene expression following CEO treatment. Differentially expressed genes (DEGs) were defined by an adjusted p value (padj) < 0.05 and an absolute log₂ fold change (|log₂FC|) ≥ 1.5. This threshold was selected to identify genes with both statistical significance and biologically meaningful expression changes. In HeLa cells, the number of upregulated (*n* = 172) and downregulated (*n* = 224) genes were comparable. In contrast, SiHa and HaCaT cells displayed predominantly upregulated DEGs, with 426 up- and 206 downregulated DEGs and 615 up- and 340 downregulated DEGs, respectively.

The Venn diagram (Fig. 7b) summarizes the unique and shared DEGs among the cell lines. HeLa, SiHa and HaCaT cells exhibited 396, 632, and 955 total DEGs, respectively, with 176, 304, and 601 genes unique to each cell line. While 36 DEGs were shared exclusively between HeLa and SiHa and 62 between HeLa and HaCaT, a large overlap of 170 DEGs was observed between SiHa and HaCaT. A core of 122 DEGs were common across all three cell lines.

Figure 7c shows a heatmap of the 98 shared DEGs that remained after excluding noncoding genes and novel transcripts from the 122 common genes identified in the Venn diagram. To prioritize genes with the strongest response, DEGs were sorted by their fold change in HeLa cells, which were the most susceptible to CEO. This analysis revealed a core set of upregulated genes common to all cell lines, including *EGR2, FOSB, CHNRA2, EGR1*, *FOS, EGR3, RASD1, ARC, RND1, FOXJ1, MAFB, and COL2A1.* Notably, the expression changes of these shared DEGs were more moderate in SiHa cells. In contrast, HaCaT cells displayed the highest expression levels of some key stress response and apoptosis regulator genes, including *HMOX1, NR4A1*, *SNAI1*, *ASGR1, and RBFOX3,* while HeLa and SiHa cells showed comparatively moderate expression of these genes.

### Cinnamon essential oil modulates pro-apoptotic, anti-proliferative, and stress-response pathways

**Fig. 8:**
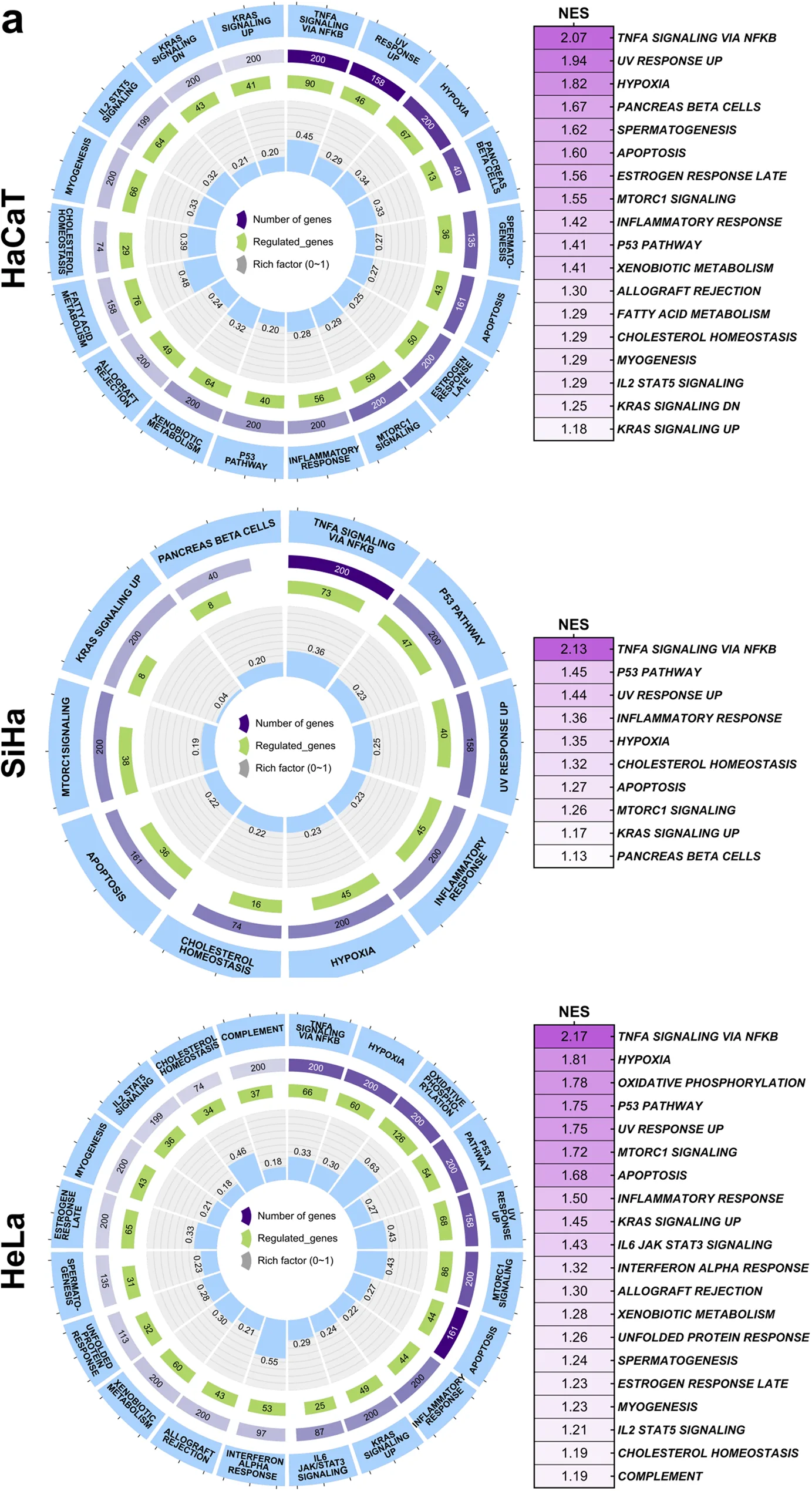

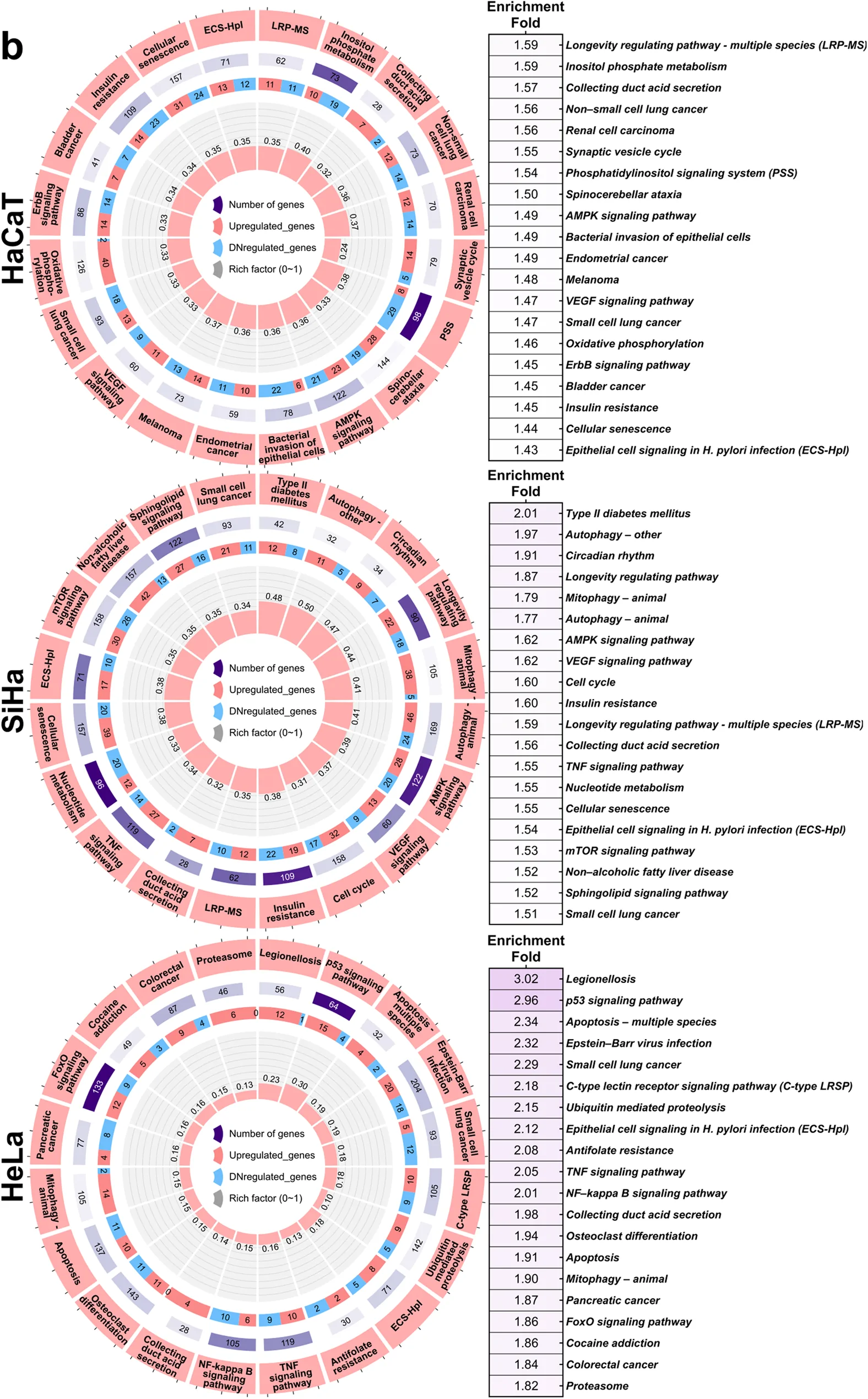
Enriched pathways from (**a**) hallmark and (**b**) KEGG pathway analyses. Polar plots illustrate the enriched pathways in the outer ring, along with the total number of genes associated with each pathway. Color intensity represents statistical significance. The subsequent ring indicates the number of enriched genes. The inner ring denotes the rich factor, calculated as the ratio of enriched genes relative to the total number of genes within the pathway. On the right, heatmaps display the NES (normalized enrichment score) in hallmark analyses or the enrichment fold for KEGG analyses. Color intensity reflects the degree of enrichment, and the numeric values within each cell indicate the enrichment score for the corresponding pathway

To gain insights into the biological and molecular pathways impacted by CEO treatment, enrichment analysis was conducted using the Hallmark (GSEA) and KEGG (via PathFindeR) databases. Hallmark analysis (Fig. 8a) identified the top 20 significantly enriched pathways across the three cell lines. The commonly enriched pathways in the most CEO-sensitive HeLa and SiHa cells were TNFα signalling via NFκB, p53 signalling, UV response up, hypoxia, apoptosis, KRAS signalling up, mTORC1 signalling, and inflammatory response.

To strengthen the enrichment analysis, KEGG pathway analysis was also performed using PathFindeR (Fig. 8b). This approach confirmed the significant involvement of several key pathways common to the response, including TNF/NF-kappa B signalling, mTOR signalling, the p53 pathway, cell cycle regulation, cellular senescence, and apoptosis. However, the enrichment of certain pathways varied across cell lines. In HaCaT cells, the most significantly enriched KEGG pathways included the longevity regulating pathway, inositol phosphate metabolism, and collecting duct acid secretion. In SiHa cells, enrichment was prominent in pathways related to Type II diabetes mellitus, Autophagy, and the Circadian rhythm and in HeLa Legionellosis, p53 signalling, and apoptosis.

### Predicted Protein‒protein Interaction (PPI) network analysis

**Fig. 9:**
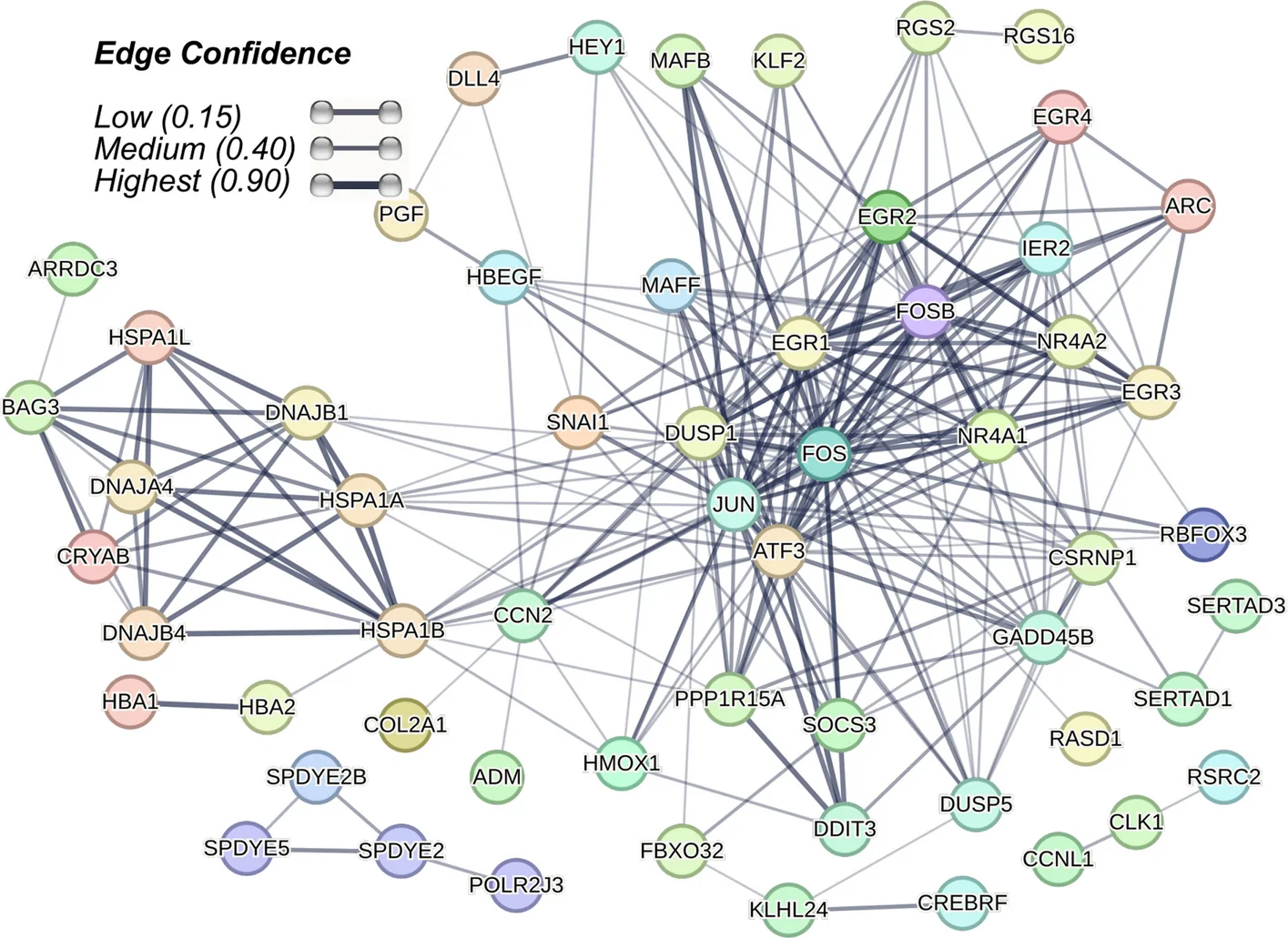
Protein–protein interaction network of the 98 filtered DEGs shared among HaCaT, SiHa, and HeLa cells. The PPI network interaction was generated using the STRING database to visualize functional associations among the gene products. It comprises 58 connected nodes, each representing a gene product, while edges denote pairwise interactions. Edge thickness reflects the confidence score of each interaction based on curated experimental data, coexpression analyses, and scientific literature

To explore functional interactions among the 98 filtered shared DEGs, protein–protein interaction (PPI) networks were constructed using the STRING database. For the subsequent protein‒protein interaction network analysis, we applied a medium confidence score (0.4) to focus on relevant nodes and excluded disconnected nodes. The resulting network (Fig. 9) revealed 58 connected nodes with three major clusters: one composed of transcriptional regulators (*FOS*, *FOSB*, *JUN*, *EGR1*, *EGR2*, *EGR3*, *DUSP1*, and *ATF3)* associated with cellular stress, proliferation, and autophagy; a second cluster containing DNA damage response genes (*GADD45B*, *SOCS3*, *DUSP5*, *DDIT3*, and *PPP1R15A)* and a putative tumor suppressor gene (*CSRNP1*); and a third cluster related to ER stress and protein folding, featuring *HSPA1A*, *HSPA1B*, *HSPA1L*, *DNAJA4*, *DNAJB1*, *DNAJB4*, *CRYAB*, and *HMOX1*. Additional file 2 further extends these findings by illustrating the PPI networks generated independently for each cell line based on their respective top 50 upregulated and downregulated genes.

**Fig. 10:**
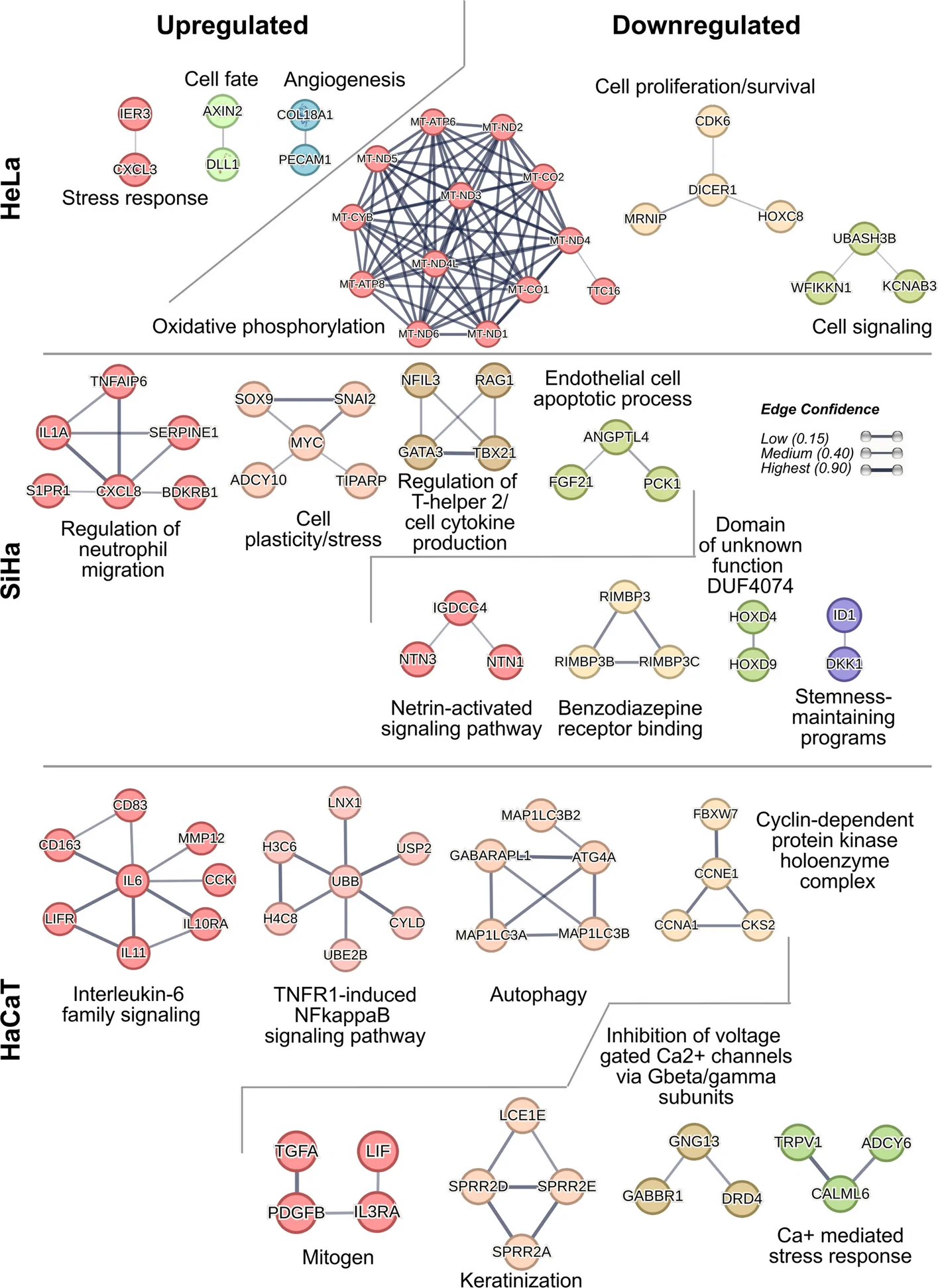
Functional clusters derived from protein–protein interaction network analysis of upregulated and downregulated genes. PPI networks were constructed from DEGs using the STRING database and analysed with the Markov cluster algorithm (MCL) to identify highly interconnected functional modules. The main clusters associated with upregulated and downregulated genes in each cell line are shown comparatively. Edge confidence levels (low = 0.15, medium = 0.40, highest = 0.90) are represented by line thickness, indicating the strength of supporting evidence for each interaction

To gain deeper insight into the mechanisms driven by CEO treatment, we determined the genes and pathways that were most significantly modified, particularly in each cell line. As shown in Fig. 10, the STRING-derived network diagram illustrates the differential up- and down- regulated biological processes and signalling pathways modulated by CEO in HeLa, SiHa, and HaCaT cells.

In HeLa cells, the primary upregulated processes were strongly associated with stress response (*CXCL3*, *IER3*), cell fate (*AXIN2*, *DLL1*) and angiogenesis (*COL18A1*, *PECAM1*), while those downregulated were linked to cell proliferation/survival (*DICER1 HOXC8*, *MRNIP*, *CDK6*) and cell signalling (*WFIKKN1*, *UBASH3B*, *KCNAB3*). Of notable interest was the substantial proportion of mitochondrial genes that were preferentially downregulated (such as *MT-CO1*, *MT-CO2*, and *MTCO2P12*).

The SiHa cell line exhibited a different regulatory profile. Upregulated pathways and genes with the highest edge confidence involved regulation of neutrophil migration (*CXCL8*, *IL1A,* and *TNFAIP6*), cell plasticity/stress (*MYC*, *SOX9*, *SNAI1*), regulation of T-helper 2/cell cytokine production (*GATA3*, *TBX21*), and endothelial cell apoptotic processes (*PCK1*, *ANGPTL4*). Conversely, the downregulated processes in SiHa cells focused on the netrin-activated signalling pathway (*NTN* −1, −3, *IGDCC4*), benzodiazepine receptor binding (*RIMBP3*, *RIMBP3B*, *RIMBP3C*), a domain of unknown function DUF4074 (*HOXD4*, *HOXD9*), and stemness-maintaining programs (*DKK1*, *ID1*).

On the other hand, in the nontumorigenic HaCaT keratinocytes, the response was distinct. One prominent cluster corresponded to interleukin-6 family signalling, centered on IL6. A second major subnetwork was enriched for components of the TNFR1-induced NF-κB signalling pathway, with UBB acting as a central hub. Additionally, a distinct cluster was associated with autophagy (*MAP1LC3A*, *ATG4A*, *GABARAPL1*, etc.), and a smaller cluster was related to the cyclin-dependent protein kinase holoenzyme complex (*CCNA1*, *CCNE1*, *CKS2*, *FBXW7*). The downregulated processes in HaCaT included mitogen (*TGFA*, *PDGFB*, *IL3RA*, *LIF*), keratinization (*LCE1E*, *SPRR2A*, *SPRR2D*, *SPRR2E*), inhibition of voltage‑gated Ca^2^⁺ channels via Gβ/γ subunits (*GABBR1*, *GNG13*, *DRD4*), and calcium‑mediated stress response (*TRPV1*, *ADCY6*, *CALML6*).

### qPCR analysis confirms the transcriptomic profile induced by cinnamon essential oil

**Fig. 11:**
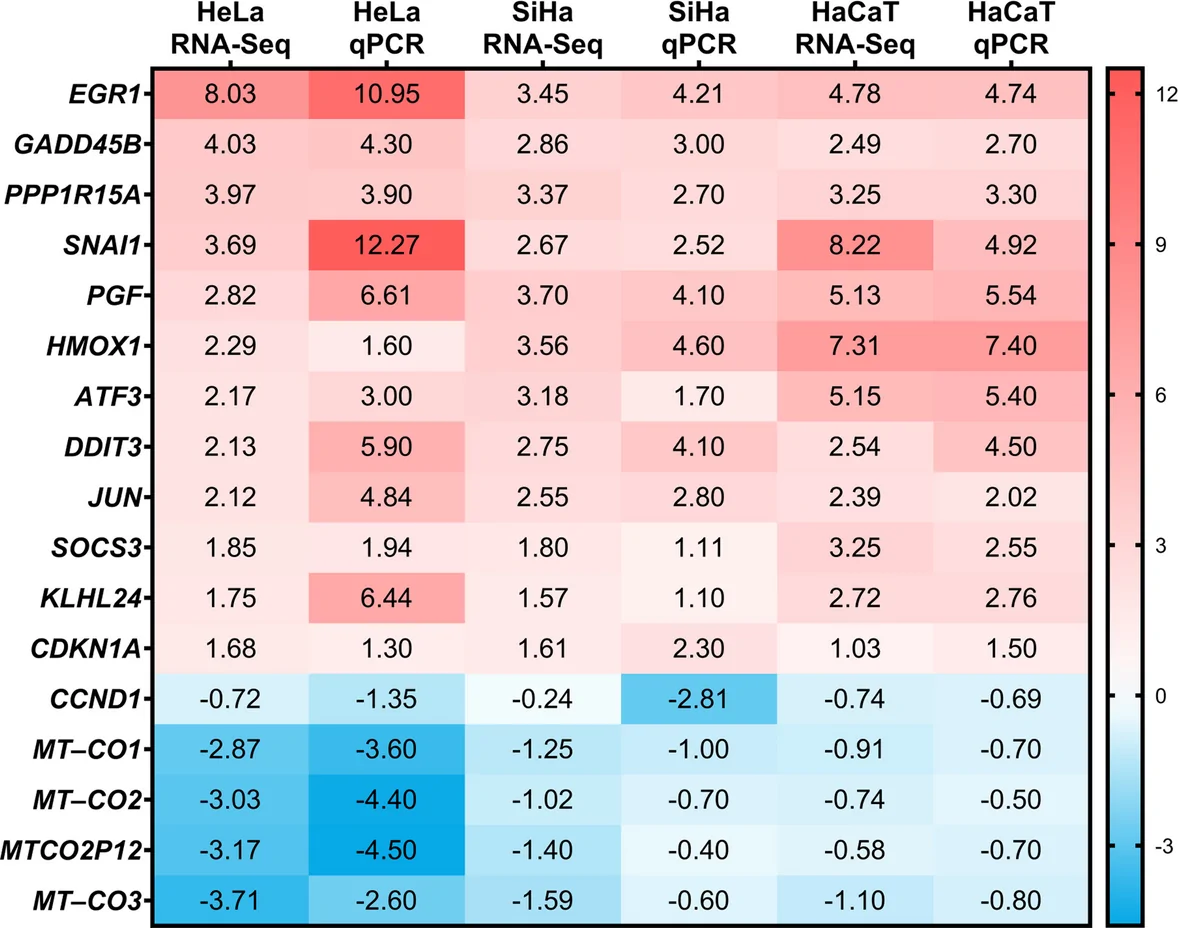
Validation of selected gene expression changes by qPCR. Heatmap displaying the expression data of 17 selected genes across HeLa, SiHa, and HaCaT cell lines following CEO treatment (1:20,000). Red boxes indicate upregulation, and blue boxes indicate downregulation, represented as Log₂FC values obtained from both RNA-seq and qPCR analyses. Gene names are listed on the right, while the top axis indicates the respective cell line and analysis type (RNA-seq or qPCR)

Finally, to validate the transcriptomic findings obtained by RNA-seq following CEO treatment, quantitative real-time PCR (qPCR) was performed on 19 selected genes from different clusters, including *EGR1*, *GADD45B*, *PPP1R15A*, *SNAI1*, *PGF*, *HMOX1*, *ATF3*, *DDIT3*, *JUN, SOCS3, KLHL24, CDKN1A*, *CCND1*, *MTCO1*, *MTCO2*, *MTCO3*, and *MTCO2P12*. As depicted in Fig. 11, the upregulation or downregulation of all selected genes was validated by these assays, with concordant trends observed between the RNA-seq and qPCR data.

## Discussion

The present study provides a comprehensive analysis of the ability of cinnamon essential oil (CEO) to induce selective cytotoxicity, mitochondrial-mediated apoptosis, suppression of HPV oncogene expression, and modulation of key transcriptomic pathways. While previous studies have demonstrated the anticancer effects of cinnamon extracts or cinnamaldehyde in various cancer models, including cervical cancer cells [44, 45], our study provides several novel contributions to the field. First, to our knowledge, this is the first comprehensive transcriptomic analysis (RNA-seq) of CEO-treated cervical cancer cells, offering a genome-wide view of the molecular pathways modulated by this natural product. Second, we demonstrate that CEO simultaneously suppresses HPV *E6*/*E7* oncogene expression while inducing mitochondrial dysfunction, a dual mechanism that has not been previously characterized in HPV-positive cervical cancer models. Third, our comparative analysis of tumorigenic (HeLa, SiHa) and nontumorigenic (HaCaT) cells yields novel insights into the selective cytotoxicity of CEO and the potential protective mechanisms in normal cells (e.g., *HMOX1* upregulation). Fourth, the identification of core transcriptional programs shared across cell lines, encompassing stress response and DNA damage pathways, establishes a molecular framework for understanding CEO's mechanism of action.

Thus, our findings demonstrate that low concentrations of CEO exert a potent and selective cytotoxic effect against cervical cancer cell lines (HeLa and SiHa), while the nontumorigenic HaCaT cells were affected to a much lesser extent, as evidenced by WST-1, Annexin V/PI, and SRB assays (Figs. 1, 2 and 3).

These results align with previous reports demonstrating the anti-neoplastic activity of cinnamon extracts against cervical cancer cells, including those observed in HeLa cells [44]. Koppikar et al. [45] reported that aqueous cinnamon extract from *Cinnamomum cassia* induced apoptosis in SiHa cells through loss of mitochondrial membrane potential. Additionally, these results are consistent with previous reports showing that CEO (*Cinnamomum zeylanicum*) preferentially targets malignant cells (MCF7, T47D, MDA-MB-231, K562, SH-SY5Y, HCT116, and fibroblasts transformed by *c-Ha-ras* oncogene 5RP7 cells) while sparing noncancerous cells, such as the human fetal lung fibroblast MRC-5 cell line or normal F2408 fibroblasts [28, 30, 46]. Moreover, *Cinnamomum zeylanicum* showed a significant chemopreventive effect in a model of chemically induced mammary carcinogenesis in rats and a significant therapeutic effect in a 4T1 adenocarcinoma model in mice [32].

The differential sensitivity observed between tumorigenic and nontumorigenic cells may reflect intrinsic differences in metabolic activity or oxidative stress responses. As has been reported in many reviews, cancer cells often exhibit higher basal levels of reactive oxygen species (ROS) and are therefore more susceptible to further oxidative insults induced by phytochemicals [47], a mechanism by which many therapies selectively kill cancer cells through ROS induction [48–50]. Furthermore, cinnamaldehyde, the main component of CEO, has been reported to trigger apoptotic signalling and/or disrupt mitochondrial function in the colon cancer cells HCT116, HT-29, SW480, and LoVo [51, 52], in the hepatocellular carcinoma HepG2 cells [53], and in the breast cancer MDA-MB-231 cells [54], which could explain the pronounced apoptotic effect in HeLa and SiHa cells.

Mechanistically, our data demonstrated that CEO-induced cell death occurs primarily through the intrinsic mitochondrial apoptotic pathway, as evidenced by significant activation of caspase-9 and loss of mitochondrial membrane potential (Δψm) measured by JC-10 (Fig. 4). These findings are consistent with previous studies reporting that cinnamon extracts or their bioactive components, such as cinnamaldehyde, can compromise mitochondrial integrity and promote cytochrome c release, ultimately leading to apoptosis. This has been observed in SiHa cells [45], the human colon cancer-derived HCT-116 cell line [55], prostate cancer-associated fibroblasts [40], human leukemia HL-60 and K562 cells [36, 56], and glioblastoma-derived U87MG cells [57]. Additionally, *Cinnamomum zeylanicum* and cinnamaldehyde have been shown to induce apoptosis in oral cancer cell lines SCC-4, SCC-9, and SCC-25 [58]. Beyond the glioblastoma model, cinnamaldehyde has been shown to act synergistically with oxaliplatin in colorectal cancer [38], with paclitaxel in breast cancer via PI3K/AKT modulation, and with cisplatin in resistant cervical cancer cells through ROS-mediated apoptosis. These findings suggest that CEO or its components could function as chemosensitizers, warranting further investigation into combination strategies for HPV-positive cervical cancer.

The EdU incorporation and SRB assays further confirmed the antiproliferative effect of CEO, with significant reductions in DNA synthesis and significant protein loss observed specifically in HeLa and SiHa cells (Figs. 2 and 3). These results are in line with other studies reporting reduced proliferation following treatment with CEO, its main component cinnamaldehyde, or other cinnamon species, such as *Cinnamomum cassia*. Using BrdU incorporation assays, similar antiproliferative effects were observed in the breast cancer-derived MCF-7 and MDA-MB-231 cell lines [32]. The relative resistance of HaCaT cells, along with their slight increase in proliferation, suggests that CEO may exert context-dependent effects based on the cellular metabolic or signalling landscape. Such selectivity could represent an advantage over conventional chemotherapeutics, which often lack discrimination between malignant and nonmalignant cells.

Notably, doxorubicin did not elicit a significant antiproliferative response in SiHa cells (Fig. 3B). This may be attributed to the differential chemosensitivity known to exist across cervical cancer cell lines at the concentrations tested (0.5–1 μM). Multiple studies have reported that SiHa cells are generally more resistant than HeLa cells to various chemotherapeutic agents: Venkatraman et al. directly demonstrated that SiHa cells were relatively more resistant to cisplatin-induced cytotoxicity than HeLa cells, showing reduced apoptotic markers and caspase activation [59]; Filippova et al. employed SiHa cells as a model of resistance to doxorubicin and cisplatin, identifying elevated antioxidant enzyme expression as a contributing mechanism [60]; and León et al. established differential sensitivity profiles across cervical cancer cell lines, including SiHa and HeLa, when exposed to doxorubicin, Taxol, and cisplatin [61]. Taken together, these findings suggest that the concentration used may have been suboptimal for SiHa cells, which may require higher doses to elicit significant cytotoxic effects.

The evaluation of cellular senescence in this study was crucial to further characterize the biological effects of CEO beyond apoptosis and proliferation arrest. Senescence is a stable cell cycle arrest that can act as a tumor-suppressive mechanism by halting the proliferation of damaged or transformed cells [62, 63]. However, persistent senescent cells may acquire a senescence-associated secretory phenotype (SASP), releasing proinflammatory cytokines, growth factors, and proteases that remodel the tissue microenvironment and can paradoxically promote tumor progression, chronic inflammation, and therapy resistance [64]. Thus, while the induction of senescence may limit immediate tumor growth, it also carries potential long-term risks [65]. In this context, we observed that CEO did not robustly induce senescence, particularly in highly sensitive HeLa cells, while doxorubicin consistently triggered senescence across all tested cell lines (Fig. 5). CEO showed only modest, cell type-dependent effects, with partial induction observed in HaCaT and SiHa cells. These findings suggest that CEO and doxorubicin inhibit cancer cell growth through distinct mechanisms. While doxorubicin primarily acts by inducing DNA breaks and inhibiting topoisomerase II activity [66], CEO may exert its effects indirectly through oxidative stress-mediated damage, potentially independently of the senescence pathway. Alternatively, the rapid induction of apoptosis could preclude senescence development in more sensitive cells such as HeLa cells. This feature is therapeutically advantageous, as it reduces the likelihood of SASP-related adverse effects and indicates that CEO eliminates cancer cells more definitively via apoptosis rather than inducing a reversible or potentially harmful latent state. Consequently, this property enhances the appeal of CEO or its bioactive components as candidates for further development into novel anticancer agents.

A particularly significant finding of our study is the downregulation of HPV oncogenes *E6* and *E7* following CEO treatment, with a more pronounced effect observed in SiHa cells (Fig. 6). These results are remarkably consistent with a very recent study published in 2025, which demonstrated that cinnamaldehyde induces degradation of both oncoproteins [67]. The alignment of our findings with this newly elucidated mechanism validates our observations and suggests that E6/E7 suppression may contribute to apoptosis in HPV-positive cervical cancer cells, alongside the mitochondrial dysfunction and stress pathway activation already discussed. It is worth noting that the transcriptional downregulation of *E6* and *E7*, while statistically significant, was relatively modest, particularly in HeLa cells. Protein-level validation would offer stronger evidence but remains technically challenging, given the well-documented limitations of commercially available antibodies for detecting endogenous E6 and E7, including poor sensitivity, cross-reactivity with cellular proteins [68], and the inherent lability and rapid turnover of the E6 protein [69, 70]. Future studies employing coimmunoprecipitation or targeted mass spectrometry-based proteomics could help overcome these limitations.

The RNA-seq analysis performed in our study provides a comprehensive overview of the global molecular response to CEO in cervical cancer cells (Figs. 7, 8, 9, and 10). The enrichment of TNFα signalling via NFκB, the p53 pathway, hypoxia, and apoptosis in hallmark analysis, together with KEGG pathway enrichment for TNF/NF-kappa B signalling, mTOR signalling, and the p53 pathway, indicates that CEO affects multiple proapoptotic and stress-responsive signalling cascades. Consistent with our observations, recent studies have confirmed that these pathways are modulated by treatment with cinnamon essential oil or its bioactive components [71, 72].

In addition, a core set of 122 common DEGs emerged across all cell lines after CEO treatment. Regarding the analysis performed with STRING (Fig. 9), prominent clusters were identified, including transcriptional regulators (*EGR1*, *EGR2*, *FOS*, *JUN*) [73, 74], stress responders (*HMOX1*, *HSPA* family, *DNAJ* family) [75–77], and genes involved in the DNA damage response (*GADD45B*, *DDIT3*, *PPP1R15A*) [78–80]*.* This profile suggests that CEO triggers broad cellular stress programs that converge on apoptosis execution specifically in cancer cells.

Concerning the individual genes found to be commonly down- or upregulated, one that particularly drew our attention was the strongly upregulated expression of *HMOX1* in the HaCaT cell line, observed consistently in both RNA-seq and qRT‒PCR validation assays (Figs. 7, 8, 9, 10 and 11). This observation aligns with reports linking cinnamaldehyde to the induction of *HMOX1* expression in human keratinocytes [81]. *HMOX1* encodes an enzyme that plays a key role in the cellular antioxidant response [82]. Given that HaCaT cells exhibited the highest resistance to CEO treatment, it is plausible that cinnamaldehyde triggered a protective antioxidant effect in these cells, which may account for their higher resistance.

Conversely, we observed a strong downregulation of *MT-CO1*, *MT-CO2*, and *MT-CO3*, particularly in HeLa cells (Fig. 11). These core components of mitochondrial complex IV [83] are essential for driving oxidative phosphorylation, the process that generates the vast majority of cellular ATP. This transcriptional repression effectively disrupts the cell´s primary energy source, aligning with the oxidative phosphorylation enrichment detected in KEGG pathways and the loss of mitochondrial membrane potential observed in functional assays. Collectively, these results indicate that CEO compromises mitochondrial bioenergetics. Additionally, trans-cinnamaldehyde has been reported to protect against oxidative stress, ROS, and mitochondrial damage in nontumorigenic C2C12 mouse cells [84]. These findings are consistent with our results in the nontumorigenic HaCaT model, where no damage was observed and minimal downregulation of mitochondrial genes was observed.

On the other hand, the downregulated genes *SLFNL1*, *MSS51*, *SPDYE2B*, *SPDYE5B*, *SPDYE5*, *SLFNL1*, *UBAP1L*, and *MARCHF1* stand out due to their key roles in cellular homeostasis. *MSS51* acts as a mitochondrial translational activator essential for respiratory chain gene expression and mitochondrial integrity [85], while members of the Speedy/RINGO family (*SPDYE2B*, *SPDYE5B*, *SPDYE5*) regulate cyclin-dependent kinase (CDK) activity, influencing cell cycle progression and the response to antiproliferative signals [86]. Together, these genes may link mitochondrial function with cell cycle control, reflecting coordinated regulation between metabolism and proliferation. This is consistent with the enrichment of oxidative phosphorylation and cell cycle pathways observed in both the hallmark and KEGG analyses. By reducing the expression of these cell cycle drivers, CEO treatment may slow or halt cell division, a key mechanism underlying its potential anticancer properties.

Although our transcriptomic analysis pointed to oxidative stress, as evidenced by the upregulation of *HMOX1* and HSPA family genes and the enrichment of TNFα/NF-κB signalling, direct measurement of ROS levels was not performed in this study. DCFH-DA-based quantification would clarify whether ROS production is a primary driver of CEO-induced cytotoxicity or a secondary consequence of mitochondrial dysfunction. Future studies incorporating both ROS quantification and antioxidant rescue experiments are warranted to further elucidate this mechanism.

Several studies have evaluated the in vivo anticancer potential of cinnamon extracts or their major bioactive component, cinnamaldehyde. Kubatka et al. [32] demonstrated that *Cinnamomum zeylanicum* bark extract exhibited significant chemopreventive efficacy in a rat model of chemically induced mammary carcinogenesis and therapeutic effects in a 4T1 adenocarcinoma mouse model. Additionally, cinnamaldehyde has been shown to inhibit tumor growth and metastasis in various xenograft models, including colorectal cancer [38], hepatocellular carcinoma, and melanoma. However, these studies are limited by variable extract compositions, lack of standardized dosing, and limited pharmacokinetic data. The translation of these findings to cervical cancer models, particularly HPV-driven tumors, remains unexplored, highlighting the need for future in vivo studies using well-characterized CEO preparations and appropriate HPV-positive tumor models.

Moreover, regarding safety, cinnamon extracts and cinnamaldehyde have generally shown favourable toxicity profiles in animal models. Oral administration of cinnamaldehyde at doses up to 500 mg/kg/day in rodents has been well tolerated, with no significant organ toxicity observed in subchronic studies [87]. In humans, although moderate consumption of cinnamon-containing foods or supplements may confer health benefits, excessive intake could trigger herb drugs via PXR or AhR activation [88], and adverse events have been documented in clinical trials [89–93], raising concerns for individuals with chronic conditions. For topical applications, essential oils may cause skin irritation at high concentrations [94]. These safety considerations underscore the importance of careful dose optimization and formulation development for potential therapeutic applications. Finally, it should be noted that the chemical composition of essential oils can vary depending on the plant source, extraction method, and batch. While we used a commercially available, standardized CEO preparation with a reported composition, independent GC‒MS characterization in future studies would enhance reproducibility and help identify the specific bioactive components responsible for the observed effects.

## Conclusion

In conclusion, CEO exhibits potent and selective antitumour activity against HPV-positive cervical cancer cells in vitro through a multifaceted mechanism that integrates the suppression of viral oncogenesis with the disruption of cellular metabolism and stress responses. Specifically, CEO triggers mitochondria-mediated apoptosis (evidenced by caspase activation and ΔΨm loss) and downregulates HPV E6/E7 oncogene expression (as confirmed by qPCR). Transcriptomic and enrichment analyses reveal robust signatures for apoptosis, oxidative phosphorylation, protein processing in the endoplasmic reticulum, and cell cycle/senescence regulation, alongside reprogramming of pathways governing proliferation, stress adaptation, and energy metabolism, including p53, TNFα/NF-κB, and mTORC1 signalling. However, further functional studies are required to confirm causality and the direct involvement of these pathways in CEO-mediated effects. The multitarget nature of CEO may offer advantages over single-target therapies. Nevertheless, it should be emphasized that these findings are derived from in vitro experiments using established cell lines. Future studies using appropriate in vivo models, including xenograft and genetically engineered mouse models, as well as primary patient-derived samples, are essential to validate the anticancer activity of CEO and its bioactive components, evaluate pharmacokinetics and bioavailability, and assess potential toxicity and therapeutic safety profiles.

Altogether, the coordinated upregulation of stress- and apoptotic-related genes, coupled with the downregulation of mitochondrial transcripts, reinforces the conclusion that CEO acts through an integrated disruption of oncogenic signalling and metabolic homeostasis, providing a strong rationale for further preclinical development.

## Supplementary Information

Supplementary Material 1.

Supplementary Material 2.

## Data Availability

The data sets generated and analyzed during the study are deposited in the Gene Expression Omnibus – NCBI (accession number GSE311189 – send on November 24, 2025 and released on May 5, 2026. Link: https://www.ncbi.nlm.nih.gov/geo/query/acc.cgi?acc=GSE311189).

## Abbreviations

AV: Annexin V
CC: Cervical cancer
CEO: Cinnamon essential oil
DEG: Differentially expressed gene
DMEM: Dulbecco's Modified Eagle Medium
DMSO: Dimethyl sulfoxide
EdU: 5-Ethynyl-2'-deoxyuridine
FBS: Foetal bovine serum
FC: Fold change
GSEA: Gene Set Enrichment Analysis
HPV: Human papillomavirus
IC_50_: Half-maximal inhibitory concentration
KEGG: Kyoto Encyclopedia of Genes and Genomes
MFI: Mean fluorescence intensity
MMP: Mitochondrial membrane potential
NES: Normalized enrichment score
PBS: Phosphate-buffered saline
PI: Propidium iodide
PPI: Protein‒protein interaction
qPCR: Quantitative polymerase chain reaction
RIN: RNA integrity number
RNA-seq: RNA sequencing
ROS: Reactive oxygen species
SASP: Senescence-associated secretory phenotype
SD: Standard deviation
SRB: Sulforhodamine B
WHO: World Health Organization
ΔΨm: Mitochondrial membrane potential
